# Discovering the Potent Inhibitors Against *Babesia bovis in vitro* and *Babesia microti in vivo* by Repurposing the Natural Product Compounds

**DOI:** 10.3389/fvets.2021.762107

**Published:** 2021-11-29

**Authors:** Yongchang Li, Mohamed Abdo Rizk, Eloiza May Galon, Mingming Liu, Jixu Li, Aaron Edmond Ringo, Shengwei Ji, Iqra Zafar, Maria Agnes Tumwebaze, Byamukama Benedicto, Naoaki Yokoyama, Ikuo Igarashi, Bayin Chahan, Xuenan Xuan

**Affiliations:** ^1^National Research Center for Protozoan Diseases, Obihiro University of Agriculture and Veterinary Medicine, Obihiro, Japan; ^2^Parasitology Laboratory, Veterinary College, Xinjiang Agricultural University, Ürümqi, China; ^3^Department of Internal Medicine and Infectious Diseases, Faculty of Veterinary Medicine, Mansoura University, Mansoura, Egypt; ^4^Department of Microbiology and Immunology, School of Basic Medicine, Hubei University of Arts and Science, Xiangyang, China; ^5^College of Agriculture and Animal Husbandry, Qinghai University, Xining, China

**Keywords:** natural product compounds, *Babesia bovis*, *in vitro*, *Babesia microti*, *in vivo*

## Abstract

In the present study, we screened 502 natural product compounds against the *in vitro* growth of *Babesia* (*B*.) *bovis*. Then, the novel and potent identified compounds were further evaluated for their *in vitro* efficacies using viability and cytotoxicity assays. The *in vivo* inhibitory effects of the selected compounds were evaluated using *B. microti* “rodent strain” in mice model. Three potent compounds, namely, Rottlerin (RL), Narasin (NR), Lasalocid acid (LA), exhibited the lowest IC_50_ (half-maximal inhibitory concentration) as follows: 5.45 ± 1.20 μM for RL, 1.86 ± 0.66 μM for NR, and 3.56 ± 1.41 μM for LA. The viability result revealed the ability of RL and LA to prevent the regrowth of treated parasite at 4 × IC_50_ and 2 × IC_50_, respectively, while 4 × IC_50_ of NR was sufficient to stop the regrowth of parasite. The hematology parameters of *B. microti in vivo* were different in the NR-treated groups as compared to the infected/untreated group. Interestingly, intraperitoneal administration of NR exhibiting inhibition in the growth of *B. microti* in mice was similar to that observed after administration of the commonly used antibabesial drug, diminazene aceturate (DA) (76.57% for DA, 74.73% for NR). Our findings indicate the richness of natural product compounds by novel potent antibabesial candidates, and the identified potent compounds, especially NR, might be used for the treatment of animal babesiosis.

## Introduction

Babesiosis is an important tick-borne disease (TBD) caused by the protozoa *Babesia* (*B*.) that infects domestic and wild animals, sometimes humans (1). In cattle, the infection is mainly caused by *B. bovis* and *B. bigemina* (2). The disease is typified by high fever, hemolytic anemia, hemoglobinuria, and occasionally death causing huge economic losses in the animal industry worldwide (1, 3).

Generally, control of babesiosis depends on three main strategies: (i) vector control, (ii) vaccine development, and (iii) administration of antibabesial drugs. One of the promising strategies against parasite is to control the receptor-ligand interactions of parasite molecules and their target cells, such as RON-AMA-1 (4). Two novel exported multigene families (mtm) that encode predicted multi-transmembrane, integral membrane proteins were identified in *B. bovis*. One mtm gene was down-regulated, resulting in decreased growth rate, reduced RBC surface ridge numbers, mis-localized VESA1, and abrogated cytoadhesion to endothelial cells (5). Furthermore, Sun et al. revealed that 70% invasion-competent *B. divergens* invading the erythrocyte were <45 s and all invasion-competent parasites achieved invasion within 10 min of contact (6). On the other hand, the commonly used antibabesial drugs, diminazene aceturate (DA) and imidocarb dipropionate (ID), exhibited resistance either from the treated parasites or toxic effect to the host (7–9), subsequently leading to discovery of safer and effective novel antibabesial compounds and becoming an urgent demand. In this regard, a non-biased screening of large libraries of compounds was recently developed to identify novel inhibitors for babesiosis (9–12). Following this pattern, Rottlerin (RL), Narasin (NR), and Lasalocid Acid (LA) are three natural compounds which were repurposing the already approved drugs. RL and NR derivative of *Mallotus philippinensis* and *salinomycin* possess multiple anti-cancer biological activities (13, 14). LA is another *ionophorous* antibiotic which also possesses anti-cancer efficacy (15). Of note, the antiparasitic efficacy of RL, NR, and LA has been reported against the growth of *Plasmodium* spp., *Eimeria* spp., *Toxoplasma gondii*, and *Trypanosoma* spp. (16–19).

RL is a polyphenol with natural anthelminthic activity isolated from *Mallotus philippinensis* in 1964 (20). In addition, RL is a polyphenol with autophagic promoting properties, and potential benefits of this compound have been identified, including anti-inflammatory, antiallergic (21), antibacterial (22), and anticancer compound (23). Furthermore, a lot of research focused on the toxicity and pharmacological mechanism of Rotterin in tumor and cancer. Moreover, RL exhibited its major and recent cytotoxic properties of human amelanotic A375 melanoma cells, that is, growth arrest, apoptosis induction, and translation shutoff. Although the RL is used in a variety of fields especially anticancer (24), as an anti-parasite, only Ietta et al. reported that RL can act against *Toxoplasma gondii*; as a result, this compound is an inducer of autophagy and inhibition of protein synthesis (25). Therefore, in the current study, we screened 502 compounds from the natural product compounds (NPCs) against the *in vitro* growth of *B. bovis* and against the *in vivo* growth of *B. microti*.

## Materials and Methods

### Parasites

*Babesia bovis* (Texas strain) was cultured in 8% purified bovine red blood cells (RBCs) suspended in GIT medium using 24-well-culture plates in a 37°C incubator with an atmospheric condition of 5% CO_2_ and 5% O_2_. The medium was replaced every 24 h (12).

*Babesia microti* (Munich strain) was used for the *in vivo* studies and was recovered from −80°C stock in one 6-week-old female BALB/c mice (CLEA Japan Inc., Tokyo, Japan) (26). The animal experiment was conducted in accordance with The Regulations for Animal Experiments of Obihiro University of Agriculture and Veterinary Medicine, Japan (Accession numbers 18–40).

### Chemical Reagents

SYBR Green I (SGI) nucleic acid stain (Lonza, USA; 10,000 ×) was stored at −30°C, and lysis buffer containing EDTA (10 mM), Tris (130 mM at pH 7.5), saponin (0.016% w/v), and TritonX-100 (1.6% v/v) was prepared and stored at 4°C, as previously described (12). Five hundred and two NPCs (2 mg/ml) (Supplementary Table 1) were received from the Cancer Research Institute of Kanazawa University (Ishikawa, Japan) and stored at −30°C until use for *in vitro* screening against *B. bovis*. DA (Novartis, Japan) was used as a positive control drug. RL, NR, and LA (all from Sigma-Aldrich, Japan) were prepared as a 100 mM stock solution and stored at −30°C until use. Cell Counting Kit-8 (CCK-8, Japan) was used for cytotoxicity assay.

### *In vitro* Growth of Initial Inhibitory Assay

The *in vitro* inhibitory efficacies of 502 NPCs were evaluated against the growth of *B. bovis* using fluorescence assay (27). All compounds were initially screened against *B. bovis* using 2.5 μg/ml at 1% parasitemia and 2.5% hematocrit (HCT) for 4 successive days in 96-well-plates. Fluorescence values were evaluated at day 4 after adding 100 μl of lysis buffer mixed with 2 × SGI using the fluorescence spectrophotometer (485 and 518 nm, Fluoroskan Ascent, USA). After the initial screening, IC_50_ values were calculated for the compounds that exhibited the highest inhibitory efficacies >60% (RL, NR, LA) with concentrations ranging from 0.10 to 100 μM using the non-linear regression analysis (Curve fit) in GraphPad Prism 7 (GraphPad Software Inc., USA). Non-parasitized RBCs and 0.50% dimethyl sulfoxide (DMSO) were loaded into triplicate wells and used as a blank and negative control, respectively. Each drug concentration was tested in triplicate, and the experiment was repeated three times.

### Viability Test and Morphological Changes Determination

The viability changes in drug-treated *B. bovis* were observed as previously described by Tayebwa et al. (28). A 96-well-plate was used, and 10 μl of 1% iRBCs with 90 μl medium at various drug concentrations were suspended in each well. The plate was incubated as aforementioned, and the medium was changed every 24 h for 4 consecutive days and replaced with their respective concentrations of RL, NR, and LA. The various concentrations of RL, NR, and LA used in this experiment were 0.50 ×, 1 ×, 2 ×, and 4 × of the IC_50_, respectively. On day 5, 3 μl of RBCs from treated wells was added to 7 μl of fresh RBCs in a new 96-well plate (no drug) and the medium was replaced daily for the next 6 days. Giemsa-stained thin blood smears (GBS) and fluorescence values were determined in 5 days. Each experiment was performed in triplicates in three separate trials.

### Cytotoxicity of RL, NR, and LA on MDCK Cell Line

The drug-exposure viability assay was performed following the recommendation of the Cell Counting Kit-8 (CCK-8, Japan). In brief, 5 × 10^4^ cells/ml of Madin-Darby Bovine Kidney (MDBK) cells were seeded on 100 μl per well in a 96-well-cell culture plate and incubated for 24 h. One hundred microliters of 3-fold drug dilutions were added to each well to a final concentration of 1–100 μM in triplicates. After 24 h, 10 μl of CCK-8 was added to each exposed drug. After 4 h incubation, the absorbance values were determined at 450 nm using MTP-500 microplate reader (Corona Electric, Japan). The wells with only the culture medium were used as blank, while those containing cells in a medium with 0.50% DMSO were used as control (29).

### Chemotherapeutic Evaluation of RL, NR and LA in Mice

The mouse model infected with *B. microti* was used to determine the inhibitory effect of the selected compounds in this study as previously described by Rizk et al. (26). Thirty-five 8-week-old female BALB/c mice (CLEA Japan Inc., Tokyo, Japan) were used in the *in vivo* study and divided equally into seven groups. First, second, and third groups were treated with RL, NR, and LA at dose rates 5 mg/kg, 7 mg/kg, and 1 mg/kg, respectively. DA (the commonly used antibabesial drug) was administrated to the mice in the fourth group at a dose rate of 25 mg/kg. Mice in the fifth group were treated with a combination therapy consisting of 3.5 mg/kg NR + 10 mg/kg DA. All drugs were administrated by intraperitoneal (IP) route in all groups. Mice in the sixth and seventh groups were kept as positive (infected and untreated) control and negative (uninfected and untreated) control, respectively. The treatment of compound was initiated and continued for 5 successive days (day 4 to day 8) when parasitemia reached 1% in the infected mice.

Prior to the beginning of the *in vivo* experiments, a *B. microti* positive mouse was prepared according to Nugraha et al. (10). In brief, *B. microti* was recovered and injected into a mouse, and then parasitemia was checked every 2 days by Giemsa-stained blood smear. After the parasitemia reached 30%, the mouse was anesthetized and blood was collected by cardiac puncture. The blood was then diluted by 1 × PBS to acquire 2 × 10^7^/ml *B. microti* iRBCs. Except the negative control group, all mice were injected IP with 0.5 ml dilution iRBC to achieve 1 x 10^7^/ml iRBCs and parasitemia was monitored every 2 days. Venous tail blood samples (2.5 μl) were collected from each mouse every 2 days until 32 days post-inoculation or the cessation of parasitemia. The blood samples were collected in a 96-well plate (RPMI 1,640 medium 100 μl + lysis buffer 50 μl), and uninfected mice RBCs were used as blank control. Then, 50 μl of lysis buffer containing 2 × SGI nucleic acid stain was added directly to each well and gently mixed (26). Next, the plate was incubated in the dark for 1 h and the fluorescence values were determined as described above using a fluorescence spectrophotometer. Meanwhile, 10 μl of blood from the tail was collected every 4 days and used to determine the hematological profiles using an automatic hemocytometer (Celltac α MEK-6,450, NihonKohden, Tokyo, Japan). All parameters were monitored until day 30. All the *in vivo* the experiments were repeated twice.

### Statistical Analysis

The IC_50_ values of RL, NR, LA, and DA were determined using the non-linear regression curve fit in GraphPad Prism 6.0 (GraphPad Software Inc., USA). The differences in the fluorescence values of the *in vitro* cultures and among groups for the *in vivo* studies were analyzed with a statistical software program (GraphPad Prism version 5.0 for Windows; GraphPad Software, Inc., San Diego, CA, USA), using an independent Student's *t*-test and one-way ANOVA. A *P-*value of < 0.05 was considered statistical difference, and a *P-*value of < 0.01 was considered statistically significant difference.

## Results

### RL, NR, and LA Inhibit the *in vitro* Growth of *B. bovis* and *in vitro* Cytotoxicity

**Figure 1:**
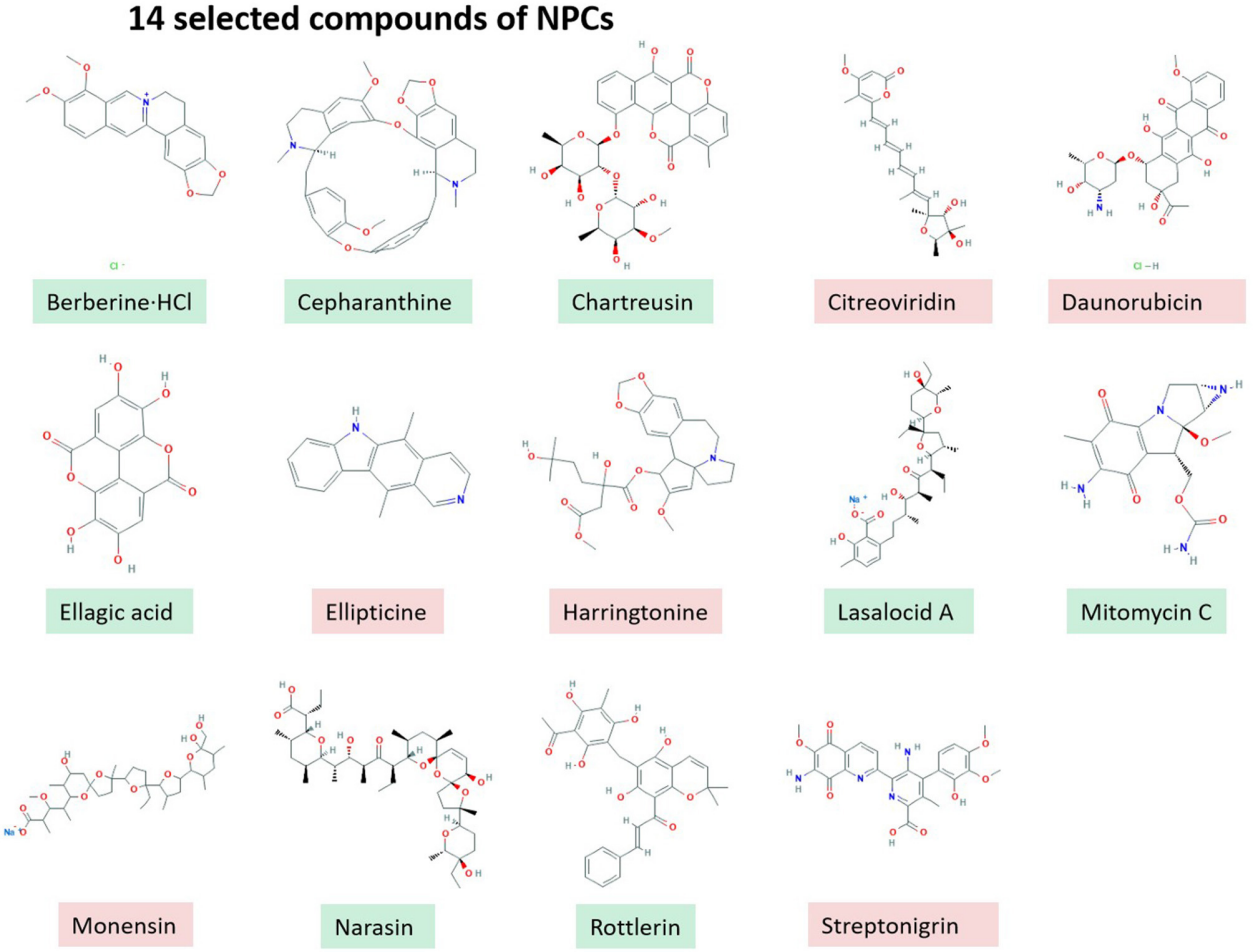
14 selected compounds of NPCs.

*In vitro* screening of 502 NPCs against the growth of *B. bovis* at 2.5 μg/ml concentration revealed that 14 compounds including RL, Berberine·HCl, Cepharanthine, Chartreusin, Citreoviridin, Daunorubicin, Ellagic acid, Ellipticine, Harringtonine, LA, Mitomycin C, Monensin, NR, and Streptonigrin exhibited over 60% inhibitory effects (Figure 1). However, cytotoxicity assay on MDBK showed that only eight compounds have low toxic effects over 100 μM (Green Color, Figure 1) and other screened compounds (*n* = 6) exhibited toxic effects (Red Color, Figure 1).

**Figure 2:**
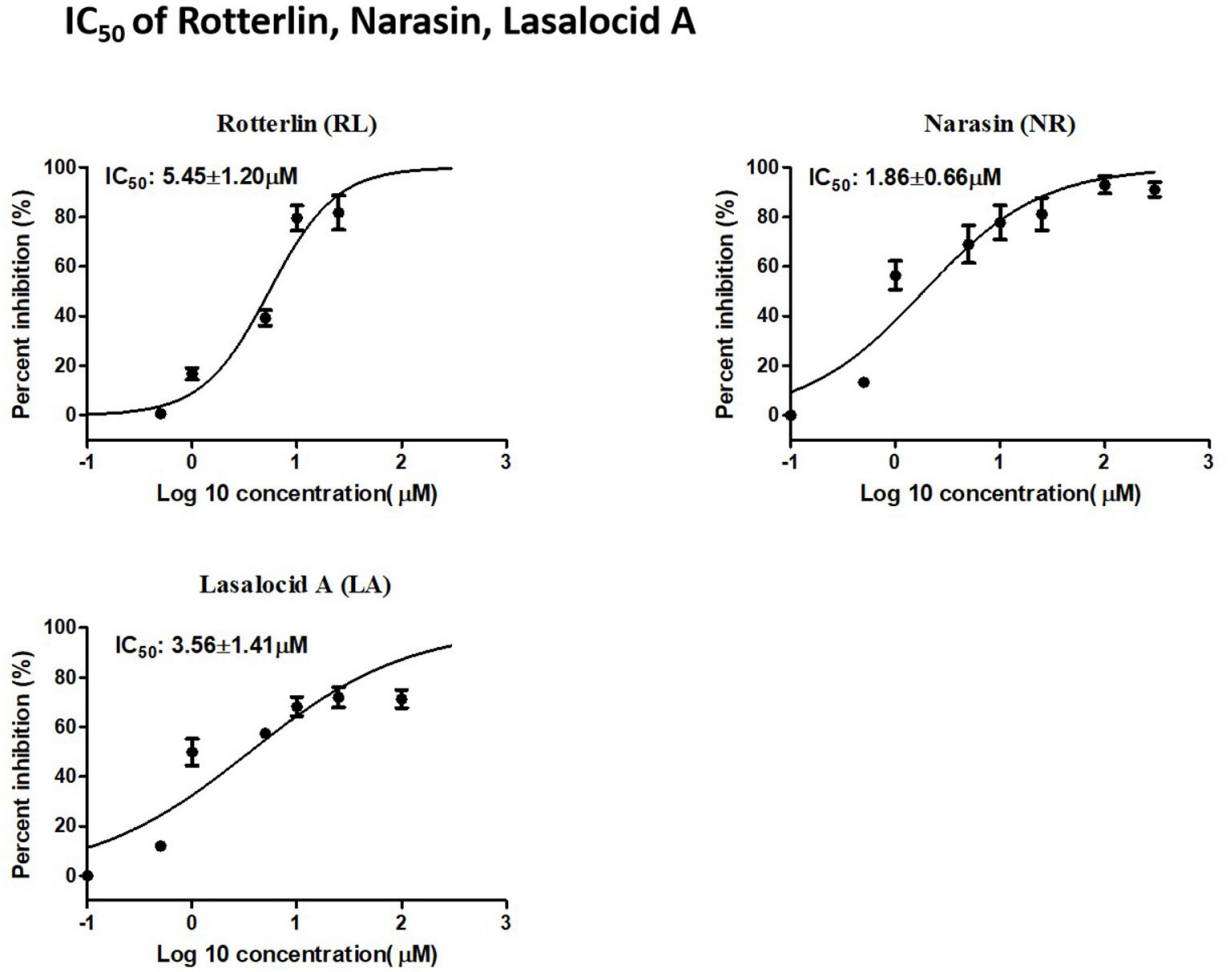
IC50 of Rotterlin, Narasin, Lasalocid A.

RL, NR, and LA inhibited the growth of *B. bovis* in a dose-dependent manner. The IC_50_ values of RL, NR, and LA were 5.45 ± 1.20 μM, 1.86 ± 0.66 μM, and 3.56 ± 1.41 μM, respectively (Figure 2).

### Viability and Morphological Changes of RL-, NR-, and LA-Treated *B. bovis*

**Figure 3:**
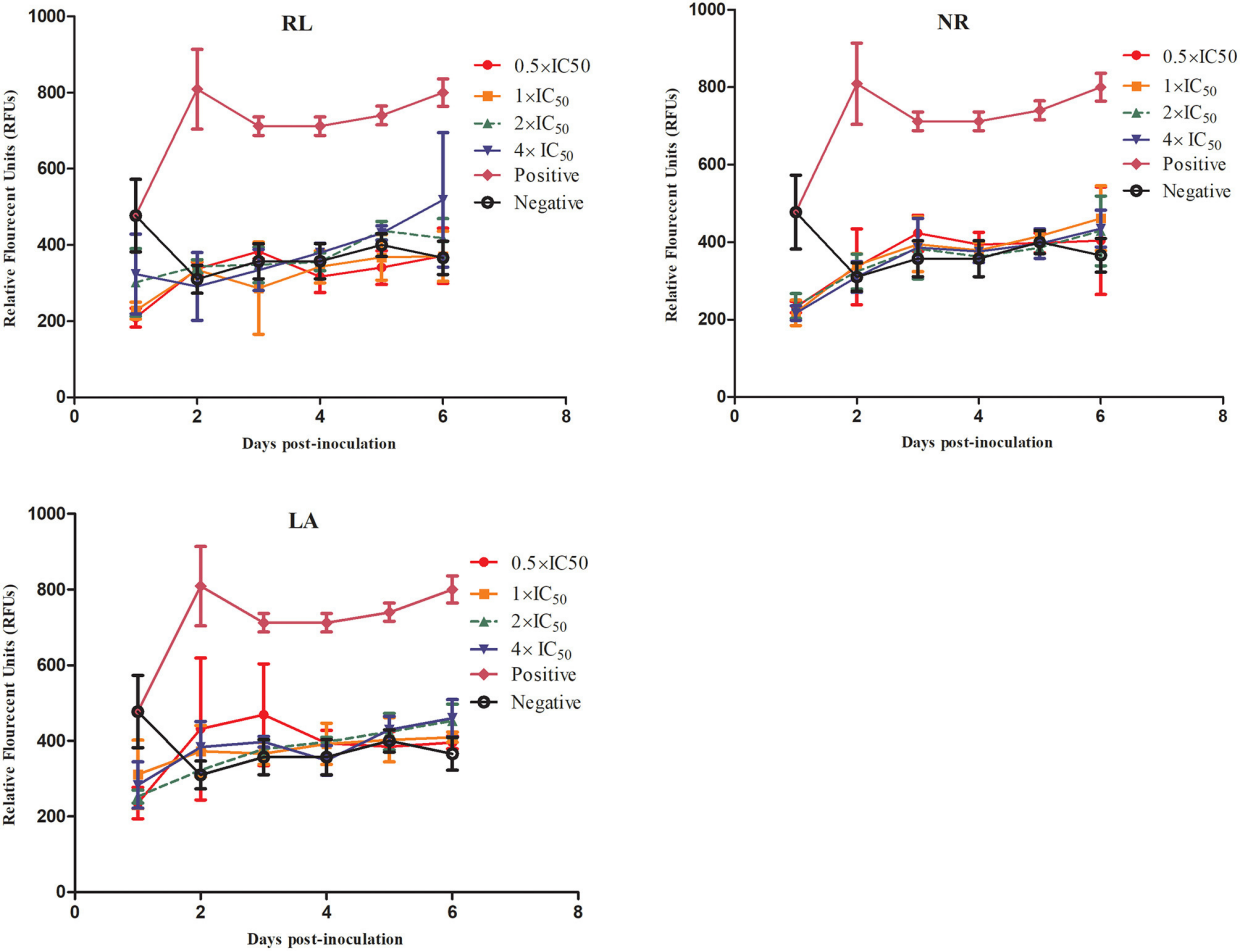
Viability test of RL, NR, and LA.

To further validate the potent identified natural product compounds as anti-*B. bovis* compounds, viability test and morphological changes in the treated culture were performed. The results showed that RL-, NR-, and LA-treated *B. bovis* lack the ability to regrow at 1 ×, 2 ×, and 4 × the IC_50_ values. The concentrations at which *B. bovis* did not regrow were 5.45, 10.90, and 21.80 μM for RL treatment, 1.86, 3.72, and 7.44 μM for NR, and 3.56, 7.12, and 14.24 μM for LA (Figure 3).

The parasitemia was examined for all parasites after 24 h, 72 h, and 7 days of incubation with the two crude methanolic extracts in Giemsa-stained blood smears. Morphological observation of *B. bovis* treated with 0.50 ×, 1 ×, 2 ×, RL, NR, and LA identified that the parasites (2 × the IC_50_) appeared smaller and disintegrated at 24 h in RL, NR, and LA as compared to the control (P3d) (Supplementary Figures 1–3). The remnants of the dot parasites within the RBC were observed in micrographs of *B. bovis* in RL, NR, and LA, while the LA-treated parasite faintly appeared at 72 h (Supplementary Figures 1–3).

### *In vitro* Cytotoxicity

*In vitro* treatment by NR exhibited no cytotoxicity until 50 μM, while the RL and LA *in vitro* treatment showed cytotoxicity on MDBK at 5 μM (Supplementary Figure 4). Fortunately, the most potent natural product compound identified, NR exhibited a low IC_50_ on MDBK with subsequently very high selectivity index (SI) (Table 1). NR was shown the highest SIs (Table 1), suggesting their possible promising future use for *in vivo* study.

**Table 1:** IC_50_, CC_50_, and selectivity indices of potent Natural compounds and diminazene aceturate evaluated against the *in vitro* growth of *B. bovis*.

**Drugs**	**IC_**50**_ values (μM)^a^**	**CC_**50**_ (μM)**	**Selectivity indices^b^**
RL	5.45 ± 1.20	27.22 ± 10.49	4.99 ± 0.68
NR	1.86 ± 0.66	95.02 ± 35.60	45.71 ± 6.12
LA	3.56 ± 0.44	9.85 ± 1.23	2.76 ± 0.11
DA	0.47 ± 0.09	ND	ND

a *IC_50_ values for each drug were calculated on the fourth day of the in vitro culture using a fluorescence assay in three separate experiments. Each drug concentration was made in triplicate in each experiment, and the final obtained IC_50_ values were the mean SD of values obtained from three separate experiments. DA, diminazene aceturate; RL, Rottlerin; NR, Narasin; LA, Lasalocid acid; ND, not detected*.

b *Selectivity indices (SIs) were calculated based on the ratio CC_50_ (MDBK)/IC_50_ of the compound*.

### Chemotherapeutic Effect of RL, NR, and LA on *B. microti* in Mice

**Figure 4:**
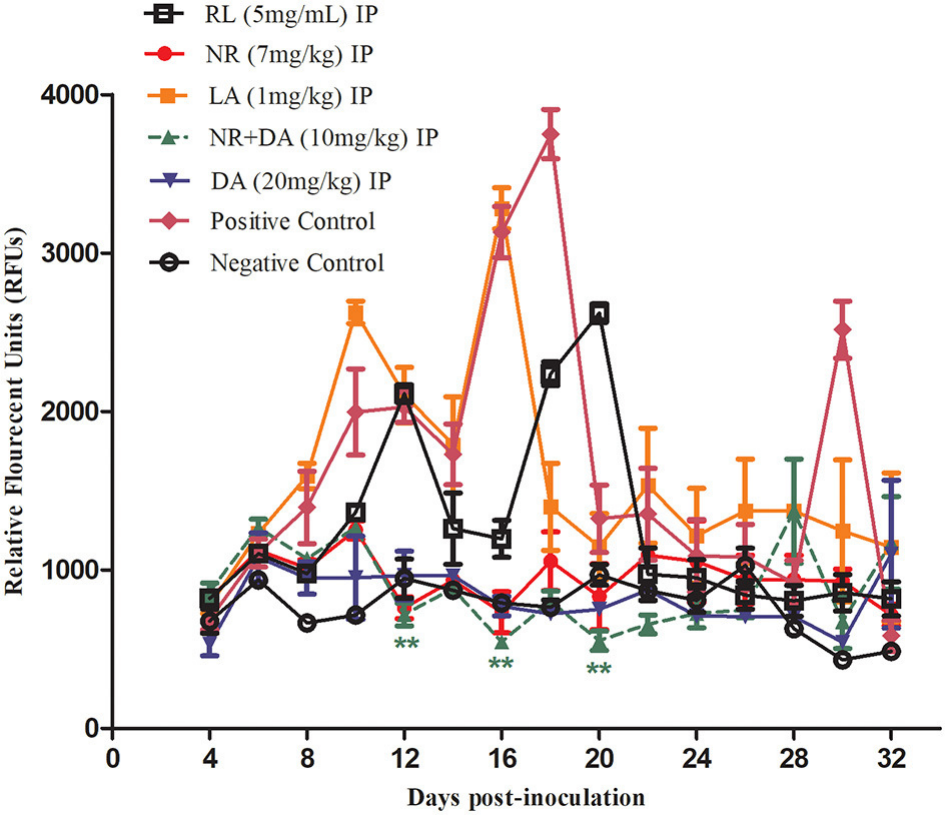
Chemotherapeutic evaluation (RFUs) of RL, NR, and LA in mice.

The promising efficacy of RL, NR, and LA *in vitro* prompted further research to evaluate the antibabesial effects against *B. microti* in mice. In treated of NR groups, the parasitemia increased at a significantly lower rate than the control group (*P* < 0.01) at days 12, 16, and 20 IP. The relative fluorescent units (RFUs) in treated groups reached 960, 1,200, 3,100, and 890 in 7 mg/kg of NR, 5 mg/kg of RL, 1 mg/kg of LA, and 20 mg/kg of DA, respectively, at 16 days IP, as compared to 3,800 peak RFUs in the control group (Figure 4). At day 16 pi, 20 mg/kg DA, 7 mg/kg NR, 5 mg/kg of RL, and 1 mg/kg LA cause 76.57, 74.73, 68.42, and 18.42% inhibition in the growth of *B. microti* in comparison with the untreated mice, respectively. Interestingly, the 5 mg/kg RL showed 68.42% inhibition in the growth of *B. microti* which is similar to those observed after treatment with the commonly used drug, DA.

**Figure 5:**
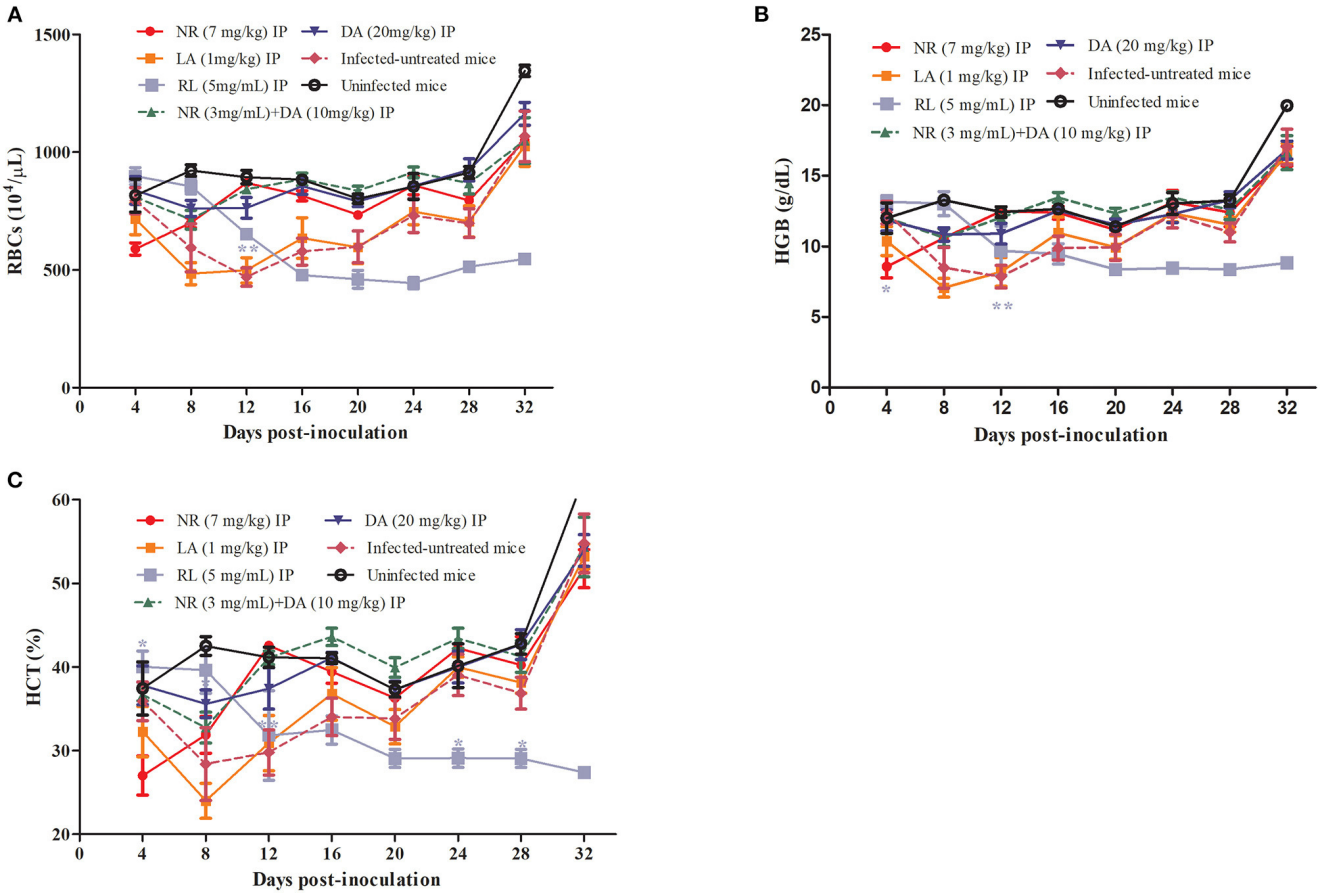
RL, NR, and LA in treatment of anemia associated with Babesia. The hematological parameters of RBCs **(A)**, HGB **(B)**, HCT **(C)**.

The hematological parameters that included the RBCs (Figure 5A), HGB concentration (Figure 5B), and HCT percentage (Figure 5C) were significantly different in the NR-treated groups as compared to the infected untreated group. On the other hand, there was no significant reduction (*P* > 0.05) in the number of RBCs, HGB concentration, and HCT percentage in the NR treated groups in comparison to the DA-treated group. The RBC, HCT, and HGB values of NR, NR+DA, and DA groups showed identical dots and line during days 8 to 16, wherein *B. microti* were having active growth. Three lines indicating RBC, HCT, and HGB were almost same as the uninfected mice, while LA did not depict much difference between infected-untreated mice. Interestingly, a decreasing trend was observed in hematological data of RL at days 0–12, but later on, low hematological parameters were shown in mice after RL was given. RL group also exhibited significant difference (*P* < 0.01) for RBCs, HCT, and HGB at day 12.

## Discussion

*Babesia bovis* is one of the most important bovine disease. So far, the vaccines against the protozoa are not very effective, but screening new effective compounds for babesiosis could be the alternative strategy for prevention and treatment of the disease. In this study, the preliminary inhibitory effects and the results of cytotoxicity assay using MDBK showed that three compounds including RL, NR, and NR exhibited the highest inhibitory efficacy against the *in vitro* growth of *B. bovis* and the lowest toxicity effect.

NR is an antibiotic to treat coccidiosis in poultry, which was isolated from *Streptomyces albus*. NR, a derivative of salinomycin (an ionophoric anticoccidial compound), and the anti-inflammatory and antiparasitic (*Plasmodium* spp. and *Eimeria* spp.) actions of NR have been previously documented (16, 18). Furthermore, NR have the inhibitory effect against the growth of *Toxoplasma gondii* (30), exhibiting over 45.56% inhibition rate when used at 1 μg/ml. According to Hickey et al. NR can also significantly reduce viable cell numbers in *Staphylococcus* aureus, and it displayed the least toxicity against mammalian cell lines including Hep G2, HFF-1, MCF-7, and MDBK (IC_50_ 39.52 μg/ml) (31). In this study, the potent inhibitory effect on *B. bovis in vitro* and *B. microti in vivo* for NR was identified. Although, this time, the data only showed the effects of natural compounds against *Babesia* pathogens, the results could be supportive for the signaling pathway found in previous scientific reports. In canine babesiosis, *Babesia rossi* has been recognized as causative agent of low triiodothyronine (T3) syndrome. The concentration of thyrotropin (TSH), total thyroxin (TT4), and free thyroxin (FT4) was found to be low in serum (32). Intriguingly, NR was identified as a small molecule antagonist for the thyroid hormone receptor beta (Thrb) in Norway rat. Thrb mediates the biological activities of thyroid hormone and is also identified in cattle and house mouse [https://pubchem.ncbi.nlm.nih.gov/gene/24831#section=Ensembl-ID, (33)]. The inhibitory effect of NR in *B. microti* (*in vivo*) may be influenced by thyroid hormone pathway. For instance, Gulia-Nuss et al. identified that the signaling pathways that regulate the innate immune response, such as the Toll-like receptors, IMD (Immunodeficiency), and JAK-STAT (Janus Kinase/ Signal Transducers and Activators of Transcription), also occur in ticks (34). Obviously, *Babesia* as one of the tick-borne pathogens can be carried and transmited by vector tick species. Gulia Nuss's investigation may explain the signaling pathway relationship for ticks and babesiosis in one aspect (34). On the other hand, Chen et al. reported that NR inhibited proliferation, migration, and invasion of human metastatic estrogen receptor positive breast cancer cells by inactivating IL-6/STAT3-mediated EMT signaling pathway*s in vitro* and *in vivo* (35).

RL is inhibitor of PKC-δ (Protein kinase C delta) gene which is also identified in human, cattle, and house mouse. PKC-δ is a tumor suppressor as well as positive regulator of cell cycle progression. Moreover, this protein can positively or negatively regulate apoptosis [https://pubchem.ncbi.nlm.nih.gov/gene/5580, (36)]. In this study, RL has the inhibitory effect on *in vitro* of *B. bovis*, but its repressing function is not well pronounced *in vivo* of *B. microti* chemotherapeutic evaluation (RFUs) in BALB/c mice. Interestingly, the identified potent NPCs prevented the development of anemia, as there was no significant reduction in the assessed hematological variables in treated mice on days p.i. The potential inhibitory effects of identified potent NPCs against *B. microti* together with the ability of these compounds to prevent the progress of anemia in the treated mice have added merit to these compounds over previously evaluated antipiroplasm candidates thymoquinone (37), fluroquinolones (38), and Zingiber officinale rhizome (39). However, further research is needed to discover the mechanism by which these compounds protect the treated mice from the hemolytic-macrocytic anemia caused by babesiosis. In some cases, the antitumor action of IFN-α has been shown to involve the induction of apoptosis through the activation of JNK *via* PKC-δ, leading to upregulation of TRAIL and activation of Stat-1. The RL as the inhibitor of PKC-δ will downregulate TRAIL and Stat-1 (40). Fascinatingly, EVs (Extracellular vesicles) from parasite or *Trypanosoma cruzi*-infected macrophages interacting with TLR2 were able to elicit translocation of NF-κB and, as a consequence, to alter the EVs, the gene expression of proinflammatory cytokines, and STAT-1 and STAT-3 signaling pathway (41). In leishmaniasis, the nuclear factor kappa-light-chain enhancer of activated B cells (NF-kB) signaling pathway and pro-apoptotic protein kinase C delta (PKC-δ) were downregulated, while inhibition of caspase-3 activation prevented *L. aethiopica* spreading (42). On the other hand, STAT-5 is also related to promote T cell proliferation and differentiation with immune-related proteins, while spleen continued to initiate immune responses to combat the infection of *B. microti* (43).

LA as a medicated feed additive is a polyether ionophore antibiotic produced by strains of the bacterium *Streptomyces lasaliensis*. This compound is used for the control of coccidiosis in cattle and poultry (44). As far as the parasite is concerned, LA reduced the growth rate of trypanosomes by 50%, and further increasing the concentration of the drug to 1.75 and 10 μM to kill the parasite. LA induced even a rapid swelling in trypanosomes than salinomycin which was considered as a trypanocidal related ionophore (45). For their anti-malarial activity, only some lasalocid acid and monensin analogs showed potent activity against selected *Plasmodium* species *in vivo* culture (14). The cyclic polyether antibiotic valinomycin and carboxylic ionophore salinomycin have been shown to exhibit potent *in vitro* anti-babesial activity against the related canine pathogen *B. gibsoni*. After using LA, the inhibitory effects of *B. bovis in vitro* were also observed. The reason of effects of lasolocid acid against *B. bovis* is probably familiar with the ionophores directly against under low concentrations of potassium (which completely lack Naþ/Kþ-ATPase activity) (45). The lysis of the red blood cells and, subsequently, the killing of *B. gibsoni* occurred when containing high concentrations of potassium (46). Unfortunately, the result of *B. microti in vivo* in BALB/c mice was not very well. Of note, polyether ionophore antibiotics have been shown to display activity against both tachyzoite and bradyzoite stages of *T. gondii* (47). On the other hand, lasalocid acid did not reduce the rate of abortion and neonatal mortality in sheep infected with *T. gondii* (48).

Although this study evaluated the inhibitory efficacy of potent compounds from the natural product against the *in vivo* growth of *B. microti*, the study neither followed up the presence of parasite remnant in different tissues of the treated mice nor determined the serum chemistry panel data before and after treatment in the mice. Therefore, additional future experiments are required to follow up the efficacy of the identified potent compounds in NPC-treated mice. This study evaluated the *in vitro* inhibitory effect of NPCs against the growth of *B. bovis* without determining the developmental stage in which the inhibition occurs. Therefore, additional future experiments are required to determine which developmental stage of the parasite might be affected by the identified NPCs, the merozoite stage outside the erythrocyte, or the parasite stage inside the erythrocyte, reducing viability or inhibiting the parasite division. In fact, the cellular targets of the identified three potent NPCs are still unclear and future studies are required to address this point.

In conclusion, new chemical compounds from natural product compounds were identified in the previous study, which suppressed the *in vitro* growth of *B. bovis* (9). But the further experiment using *B. microti* in BALB/c mice showed the toxic effects in mice using natural product compounds, and NR from the three selected compounds was found to have good inhibitory effect whether *in vitro* of *B. bovis* and *in vivo* of *B. microti* in BALB/c mice. Our findings indicated that natural product compounds are a precious source for discovering novel antibabesial drugs and the identified potent compounds, especially NR that might be used for the treatment of Babesiosis.

## Data Availability Statement

The original contributions presented in the study are included in the article/Supplementary Material, further inquiries can be directed to the corresponding author/s. And I detected the following words and expressions: Global list: https://pubchem.ncbi.nlm.nih.gov/, ncbi.nlm.nih.gov/, PubChem, pubchem.ncbi.nlm.nih.gov/.

## Ethics Statement

The animal experiment was conducted in accordance with the Regulations for Animal Experiments of Obihiro University of Agriculture and Veterinary Medicine, Japan (Accession No. 18–40).

## Author Contributions

YL: methodology, validation, statistical analysis of the results, writing—original draft, and writing—review and editing. MR: methodology, validation, statistical analysis of the results, and writing—review and editing. EG, ML, and JL: validation and writing—review and editing. AR, SJ, and IZ: recorded samples' data and writing—review and editing. MT and BB: writing—review and editing. NY and II: methodology. BC: writing—review and editing and funding acquisition. XX: conceptualization, writing—review and editing, and funding acquisition. All authors contributed to the article and approved the submitted version.

## Funding

This study was supported by grants from the Japan Society for the Promotion of Science (JSPS) Core to Core Program, Shanghai Cooperation Organization of Xinjiang Uygur Autonomous Region (No. 2021E01001), and Postdoctoral program of Xinjiang Agricultural University, Xinjiang Uygur Autonomous Region.

## Conflict of Interest

The authors declare that the research was conducted in the absence of any commercial or financial relationships that could be construed as a potential conflict of interest.

## Publisher's Note

All claims expressed in this article are solely those of the authors and do not necessarily represent those of their affiliated organizations, or those of the publisher, the editors and the reviewers. Any product that may be evaluated in this article, or claim that may be made by its manufacturer, is not guaranteed or endorsed by the publisher.

## Supplementary Material

Supplementary Table 1 Natural compounds BML-2865 information.

Supplementary Figure 1 Morphological changes observed in 0.5 × -2 × Rotterolin treated *B. bovis*.

Supplementary Figure 2 Morphological changes observed in 0.5 × -2 × Narasin treated *B. bovis*.

Supplementary Figure 3 Morphological changes observed in 0.5 × -2 × Lasalocid A treated *B. bovis*.

Supplementary Figure 4 Cytotoxicity of RL, NR, and LA in MDBK.

## References

[1] Uilenberg G. Babesia–a historical overview. Vet Parasitol. (2006) 138:3–10. 10.1016/j.vetpar.2006.01.035 16513280

[2] Keroack CD Elsworth B Duraisingh MT. To kill a piroplasm: genetic technologies to advance drug discovery and target identification in Babesia. Int J Parasitol. (2018) 49:153–63. 10.1016/j.ijpara.2018.09.005 30391230

[3] Suarez CE Alzan HF Silva MG Rathinasamy V Poole WA Cooke BM. Unravelling the cellular and molecular pathogenesis of bovine babesiosis: is the sky the limit. Int J Parasitol. (2019) 49:183–97. 10.1016/j.ijpara.2018.11.002 30690089 PMC6988112

[4] Cuy-Chaparro L Bohórquez MD Arévalo-Pinzón G Castañeda-Ramírez JJ Suárez CF Pabón L. *Babesia bovis* ligand-receptor interaction: AMA-1 contains small regions governing bovine erythrocyte binding. Int J Mol Sci. (2021) 13:22. 10.3390/ijms22020714 33450807 PMC7828228

[5] Hakimi H Templeton TJ Sakaguchi M Yamagishi J Miyazaki S Yahata K. Novel *Babesia bovis* exported proteins that modify properties of infected red blood cells. PLoS Pathog. (2020) 10:16. 10.1371/journal.ppat.1008917 33017449 PMC7561165

[6] Sun Y Moreau E Chauvin A Malandrin L. The invasion process of bovine erythrocyte by *Babesia divergens* knowledge from an *in vitro* assay. Vet Res. (2011) 42:62. 10.1186/1297-9716-42-62 21569363 PMC3117698

[7] Hwang SJ Yamasaki M Nakamura K Sasaki N Murakami M Rajapakshage BK. Development and characterization of a strain of *Babesia gibsoni* resistant to diminazene aceturate *in vitro*. J Vet Med Sci. (2010) 72:765–71. 10.1292/jvms.09-0535 20160418

[8] Mosqueda J Olvera-Ramirez A Aguilar-Tipacamu G Canto GJ. Current advances in detection and treatment of babesiosis. Curr Med Chem. (2012) 19:1504–18. 10.2174/092986712799828355 22360483 PMC3355466

[9] Rizk MA El-Sayed SAE Nassif M Mosqueda J Xuan X Igarashi I. Assay methods for *in vitro* and *in vivo* anti-Babesia drug efficacy testing: current progress, outlook, and challenges. Vet Parasitol. (2020) 279:109013. 10.1016/j.vetpar.2019.109013 32070899

[10] Nugraha AB Tuvshintulga B Guswanto A Tayebwa DS Rizk MA Gantuya S. Screening the medicines for malaria venture pathogen box against piroplasm parasites. Int J Parasitol Drugs Drug Resist. (2019) 10:84–90. 10.1016/j.ijpddr.2019.06.004 31254719 PMC6603297

[11] Rizk MA El-Sayed SAE El-Khodery S Yokoyama N Igarashi I. Discovering the *in vitro* potent inhibitors against *babesia* and *theileria* parasites by repurposing the malaria box: a review. Vet Parasitol. (2019) 274:108895. 10.1016/j.vetpar.2019.07.003 31494399

[12] Li Y Liu M Rizk MA Moumouni PFA Lee SH Galon EM. Drug screening of food and drug administration-approved compounds against *Babesia bovis in vitro*. Exp Parasitol. (2020) 52:371–8. 10.1016/j.exppara.2020.107831 31926147

[13] Ma J Hou Y Xia J Zhu X Wang ZP. Tumor suppressive role of rottlerin in cancer therapy. Am J Transl Res. (2018) 10:3345–56. eCollection 2018. 30662591 PMC6291697

[14] Markowska A Kaysiewicz J Markowska J Huczyński A. Doxycycline, salinomycin, monensin and ivermectin repositioned as cancer drugs. Bioorg Med Chem Lett. (2019) 29:1549–54. 10.1016/j.bmcl.2019.04.045 31054863

[15] Sulik M Maj E Wietrzyk J Huczyński A Antoszczak M. Synthesis and anticancer activity of dimeric polyether ionophores. Biomolecules. (2020) 10:1039. 10.3390/biom10071039 32664671 PMC7408349

[16] Gerhold RW Fuller AL Lollis L Parr C McDougald LR. The efficacy of anticoccidial products against *Eimeria spp*. in Northern Bobwhites. Avian Dis. (2011) 55:59–64. 10.1637/9572-101310-Reg.1 21500637

[17] Gumila C Ancelin ML Delort AM Jeminet G Vial HJ. Characterization of the potent *in vitro* and *in vivo* antimalarial activities of ionophore compounds, Antimicrob. Antimicrob Agents Chemother. (1997) 41:523–9. 10.1128/AAC.41.3.523 9055986 PMC163744

[18] Kevin Ii DA Meujo DA Hamann MT. Polyether Ionophores: Broad-spectrum and promising biologically active molecules for the control of drug-resistant bacteria and parasites. Expert Opin Drug Discov. (2009) 4:109–46. 10.1517/17460440802661443 23480512 PMC4896753

[19] Steverding D Huczyński A. *Trypanosoma brucei*: trypanocidal and cell swelling activities of lasalocid acid. Parasitol Res. (2017) 116:3229–33. 10.1007/s00436-017-5624-6 28956164 PMC5660140

[20] Gupta SS Verma P Hishikar K. Purgative and anthelmintic effects of Mallotus philippinensis in rats against tape worm. Indian J Physiol Pharmacol. (1984) 28:63–6. 6490133

[21] Ishii R Horie M Saito K Arisawa M Kitanaka S. Prostaglandin E(2) production and induction of prostaglandin endoperoxide synthase-2 is inhibited in a murine macrophage-like cell line, RAW 264.7, by Mallotus japonicus phloroglucinol derivatives. Biochim Biophys Acta. (2002) 1571:115–23. 10.1016/S0304-4165(02)00200-3 12049791

[22] Zaidi SFH Yoshida I Butt F Yusuf MA Usmanghani K Kadowaki M. Potent bactericidal constituents from Mallotus philippinensis against clarithromycin and metronidazole resistant strains of Japanese and Pakistani helicobacter pylori. Biol Pharm Bull. (2009) 32:631–6. 10.1248/bpb.32.631 19336896

[23] Maioli E Torricelli C Valacchi G. Rottlerin and cancer: novel evidence and mechanisms. Sci World J. (2012) 2012:350826. 10.1100/2012/350826 22272173 PMC3259573

[24] Ietta F Valacchi G Benincasa L Pecorelli A Cresti L Maioli E. Multiple mechanisms of Rottlerin toxicity in a375 melanoma cells. Biofactors. (2019) 45:920–9. 10.1002/biof.1551 31408224

[25] Ietta F Maioli E Daveri E Oliveira JG da Silva RJ Romagnoli R. Rottlerin-mediated inhibition of *Toxoplasma gondii* growth in BeWo trophoblast-like cells. Sci Rep. (2017) 7:1279. 10.1038/s41598-017-01525-6 28455500 PMC5430667

[26] Rizk MA El-Sayed SA AbouLaila M Eltaysh R Yokoyama N Igarashi I. Performance and consistency of a fluorescence-based high-throughput screening assay for use in Babesia drug screening in mice. Sci Rep. (2017) 7:12774. 10.1038/s41598-017-13052-5 29038534 PMC5643553

[27] Rizk MA El-Sayed SAES Terkawi MA Youssef MA El Said ESES Elsayed G. Optimization of a fluorescence-based assay for large-scale drug screening against babesia and theileria parasites. PLoS ONE. (2010) 10:0125276. 10.1371/journal.pone.0125276 25915529 PMC4411034

[28] Tayebwa DS Tuvshintulga B Guswanto A Nugraha AB Batiha GE Gantuya S. The effects of nitidine chloride and camptothecin on the growth of *Babesia* and *Theileria* parasites. Ticks Tick Borne Dis. (2018) 9:1192–1201. 10.1016/j.ttbdis.2018.04.019 29730263

[29] Baneth G. Antiprotozoal treatment of canine babesiosis. Vet Parasitol. (2008) 254:58–63. 10.1016/j.vetpar.2018.03.001 29657012

[30] Adeyemi OS Atolani O Awakan OJ Olaolu TD Nwonuma CO Alejolowo O. *In vitro* screening to identify anti-toxoplasma compounds and in silico modeling for bioactivities and toxicity. Yale J Biol Med. (2019) 92:369–83. eCollection 2019 Septtember. 31543702 PMC6747942

[31] Hickey EE Wong HS Khazandi M Ogunniyi AD Petrovski KR Garg S. Repurposing ionophores as novel antimicrobial agents for the treatment of bovine mastitis caused by gram-positive pathogens. J Vet Pharmacol Ther. (2018) 41:746–54. 10.1111/jvp.12674 29971788

[32] Zygner W Gójska-Zygner O Baska P Długosz E. Low t3 syndrome in canine babesiosis associated with increased serum il-6 concentration and azotaemia. Vet Parasitol. (2015) 30:211. 10.1016/j.vetpar.2015.04.023 25976636

[33] National library of medicine National Center for Biotechnology Information. Thrb – Thyroid Hormone Receptor Beta (Norway rat). (2021). Available online at: https://pubchem.ncbi.nlm.nih.gov/gene/24831#section=Ensembl-ID (accessed October 16, 2021).

[34] Gulia-Nuss M Nuss AB Meyer JM Sonenshine DE Roe RM Waterhouse RM. Genomic insights into the *Ixodes scapularis* tick vector of Lyme disease. Nat Commun. (2016) 7:10507. 10.1038/ncomms10507 26856261 PMC4748124

[35] Chen J Huang X Li N Liu B Ma Z Ling J. Narasin inhibits tumor metastasis and growth of ERα-positive breast cancer cells by inactivation of the TGF-β/SMAD3 and IL-6/STAT3 signaling pathways. Mol Med Rep. (2020) 22:5113–24. 10.3892/mmr.2020.11624 33174044 PMC7646975

[36] National library of medicine National Center for Biotechnology Information. PRKCD - Protein Kinase C Delta (Cattle, Norway Rat). (2021). Available online at: https://pubchem.ncbi.nlm.nih.gov/gene/5580 (accessed October 16, 2021).

[37] El-Sayed SAE Rizk MA Yokoyama N Igarashi I. Evaluation of the *in vitro* and *in vivo* inhibitory effect of thymoquinone on piroplasm parasites. Parasit Vectors. (2019) 12:37. 10.1186/s13071-019-3296-z 30651142 PMC6335684

[38] Rizk MA AbouLaila M El-Sayed SA Guswanto A Yokoyama N Igarashi I. Inhibitory effects of fluoroquinolone antibiotics on *Babesia divergens* and *Babesia microti*, blood parasites of veterinary and zoonotic importance. Infect Drug Resist. (2018) 11:1605–15. 10.2147/IDR.S159519 30310296 PMC6166754

[39] Rizk MA El-Sayed SAE Igarashi I. Evaluation of the inhibitory effect of Zingiber officinale rhizome on Babesia and Theileria parasites. Parasitol Int. (2021) 85:102431. 10.1016/j.parint.2021.102431 34352378

[40] Vega GG Franco-Cea LA Huerta-Yepez S Mayani H Morrison SL Bonavida B. Overcoming rituximab drug-resistance by the genetically engineered anti-CD20-hIFN-α fusion protein: direct cytotoxicity and synergy with chemotherapy. Int J Oncol. (2015) 47:1735–48. 10.3892/ijo.2015.3170 26398317 PMC4735703

[41] Cronemberger-Andrade A Xander P Soares RP Pessoa NL Campos MA Ellis CC. Trypanosoma cruzi-infected human macrophages shed proinflammatory extracellular vesicles that enhance host-cell invasion *via* toll-like receptor 2. Front Cell Infect Microbiol. (2020) 10:99. 10.3389/fcimb.2020.00099 32266161 PMC7098991

[42] Ranatunga M Rai R Richardson SCW Dyer P Harbige L Deacon A. *Leishmania aethiopica* cell-to-cell spreading involves caspase-3, AkT, and NF-κB but not PKC-δ activation and involves uptake of LAMP-1-positive bodies containing parasites. FEBS J. (2020) 287:1777–97. 10.1111/febs.15166 31804757

[43] Xue X Ren S Yang X Masoudi A Hu Y Wang X. Protein regulation strategies of the mouse spleen in response to *Babesia microti* infection. Parasit Vectors. (2021) 14:61. 10.1186/s13071-020-04574-5 33468223 PMC7814643

[44] Noack S Chapman HD Selzer PM. Anticoccidial drugs of the livestock industry. Parasitol Res. (2019) 118:2009–26. 10.1007/s00436-019-06343-5 31152233 PMC6611755

[45] Osorio Y Travi BL Renslo AR Peniche AG Melby PC. Identification of small molecule lead compounds for visceral leishmaniasis using a novel *ex vivo* splenic explant model system. PLoS Negl Trop Dis. (2011) 5:e962. 10.1371/journal.pntd.0000962 21358812 PMC3039689

[46] Yamasaki M Nakamura K Tamura N Hwang SJ Yoshikawa M Sasaki N. Effects and mechanisms of action of ionophorous antibiotics valinomycin and salinomycin-Na on *Babesia gibsoni in vitro*. J Parasitol. (2009) 95:1532–8. 10.1645/GE-2036.1 20929429

[47] Couzinet S Dubremetz JF Buzoni-Gatel D Jeminet G Prensier G. *In vitro* activity of the polyether ionophorous antibiotic monensin against the cyst form of *Toxoplasma gondii*. Parasitology. (2000) 121:359–65. 10.1017/S0031182099006605 11072898

[48] Kirkbride CA Dubey JP Libal MC. Effect of feeding lasalocid to pregnant ewes experimentally infected with *Toxoplasma gondii*. Vet Parasitol. (1992) 44:299–303. 10.1016/0304-4017(92)90126-T 1466138

