# Supplementary material for: Discovering the Potent Inhibitors Against Babesia bovis in vitro and Babesia microti in vivo by Repurposing the Natural Product Compounds
Source: Front Vet Sci. 2021 Nov 29;8:762107. doi: 10.3389/fvets.2021.762107 (PMC8666878; doi:10.3389/fvets.2021.762107)
Supplement: Supplementary file 1 [file Data_Sheet_1.pdf]

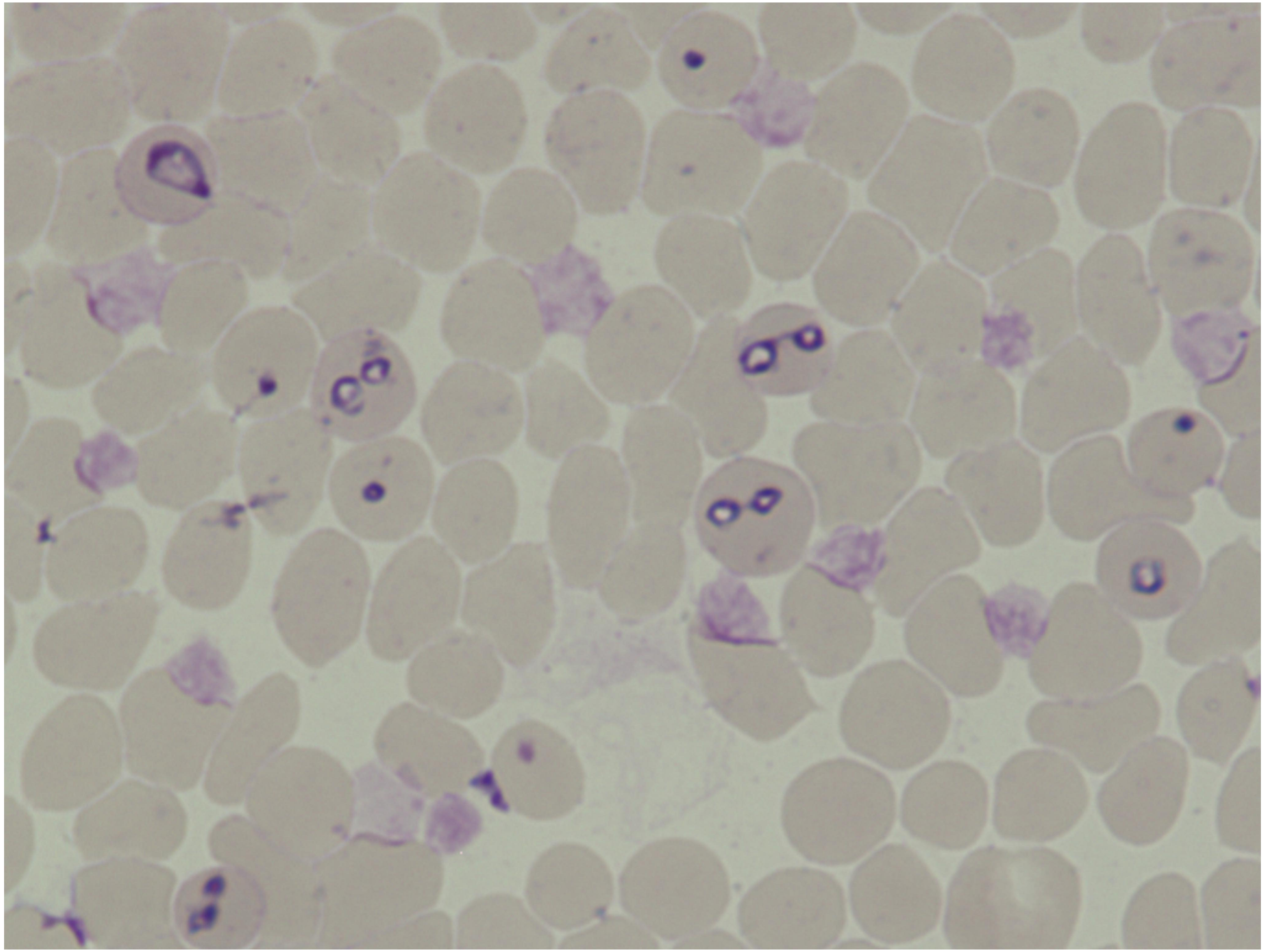

Figure S1
Positive P3d
（200×10）

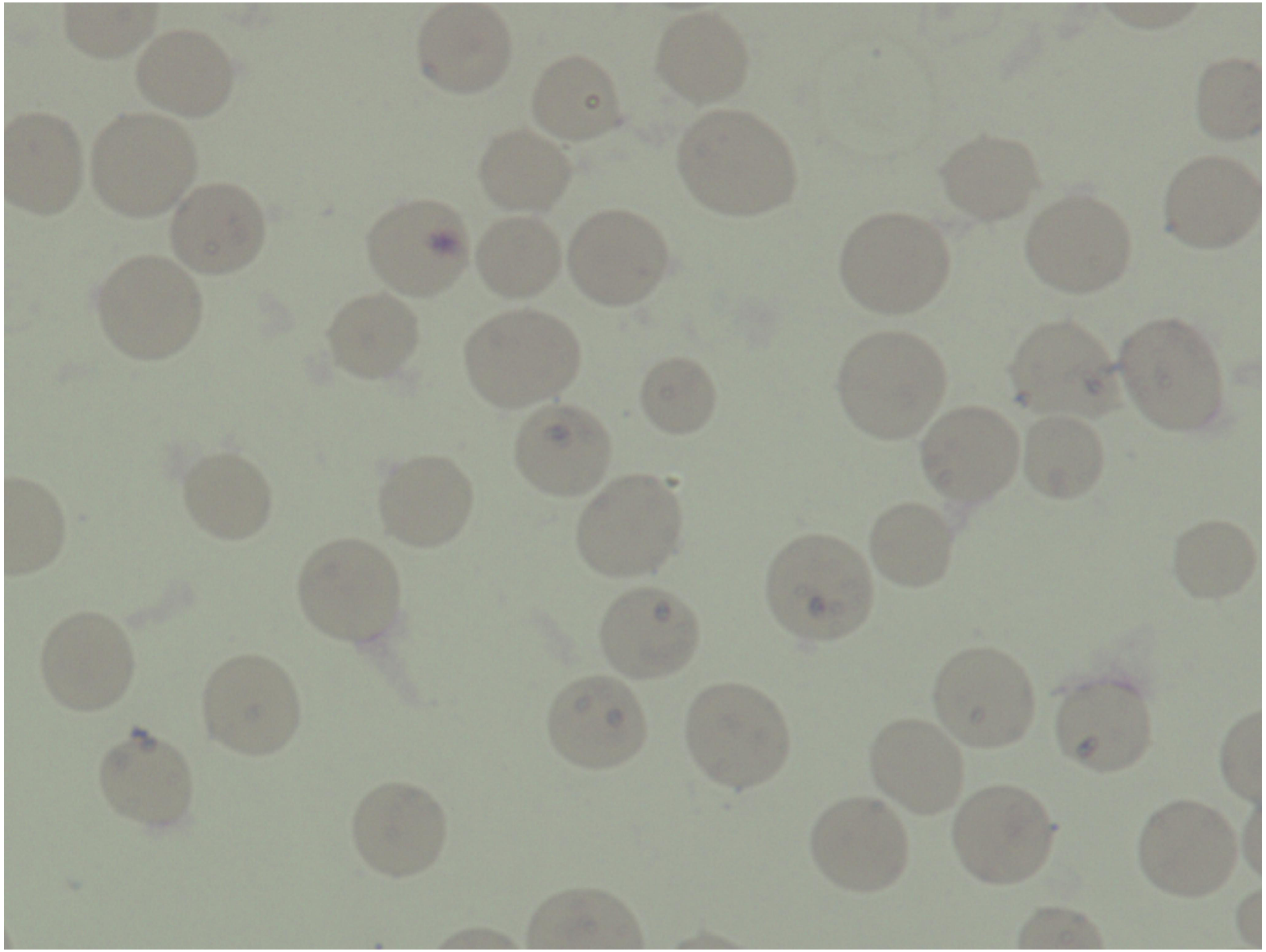

Figure S1 0.5x RO
24h（200×10）

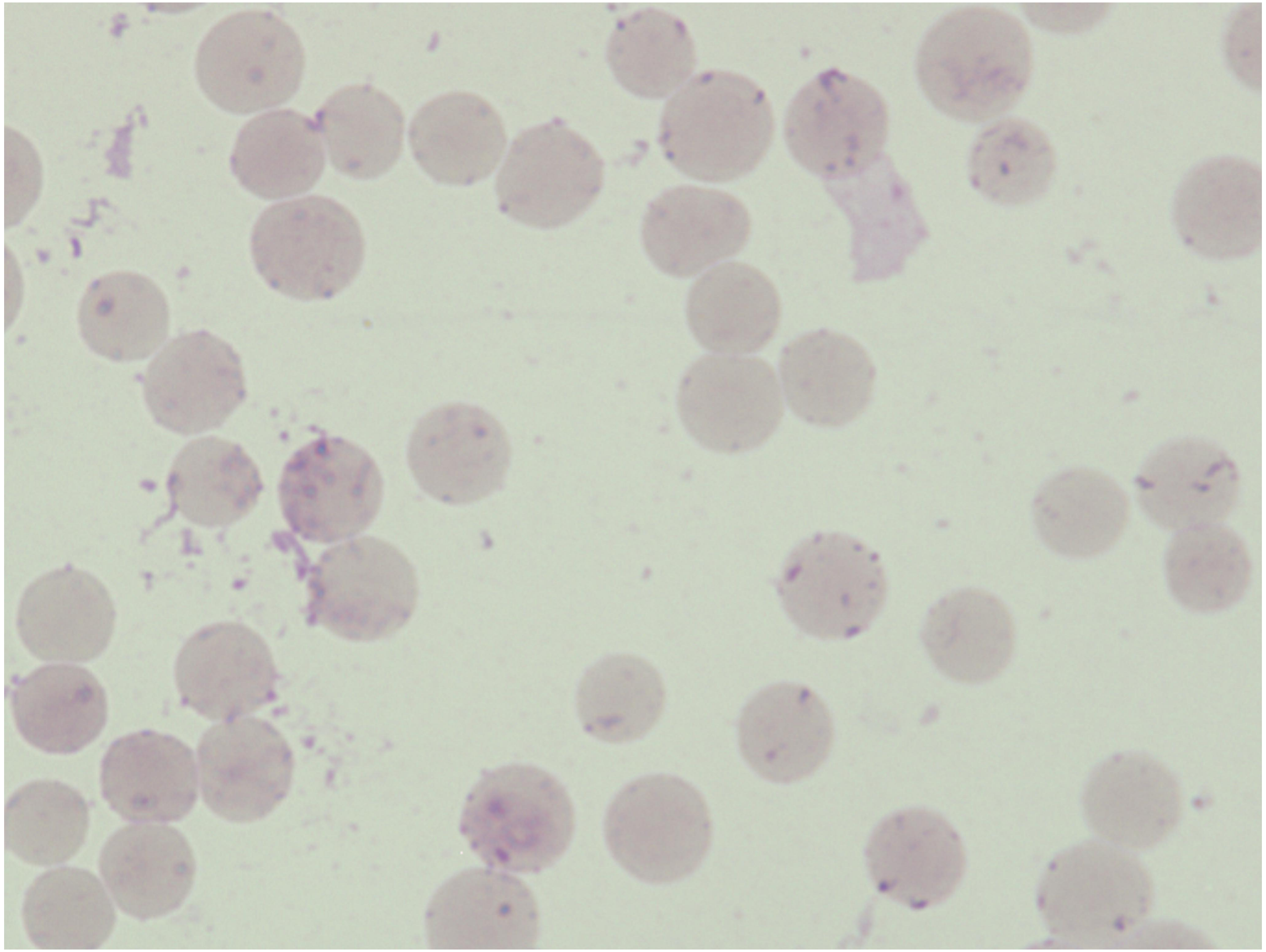

Figure S1 0.5x RO
72h （200×10）

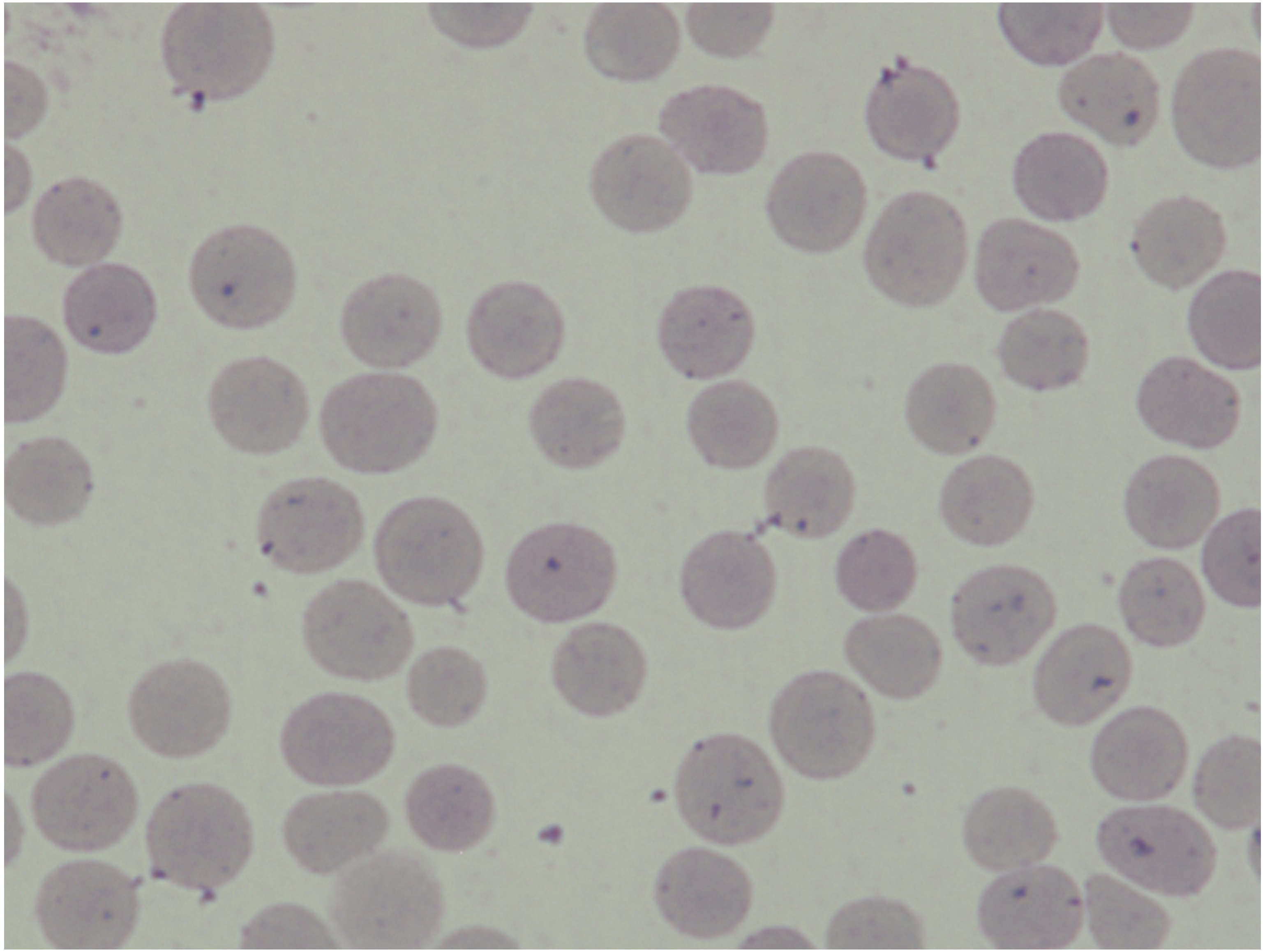

Figure S1 0.5x RO
7d （200×10）

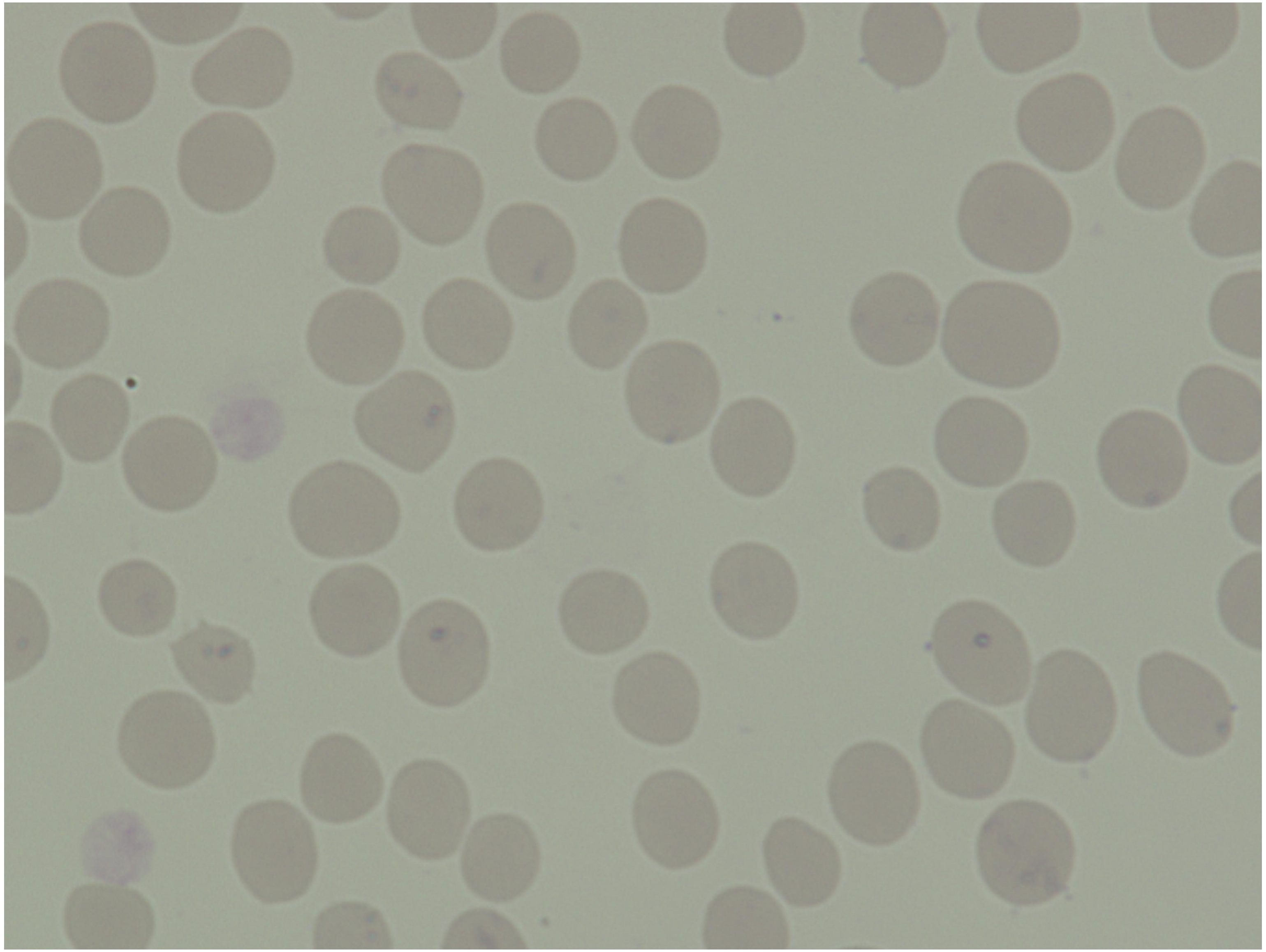

Figure S1 1x RO 24h
（200×10）

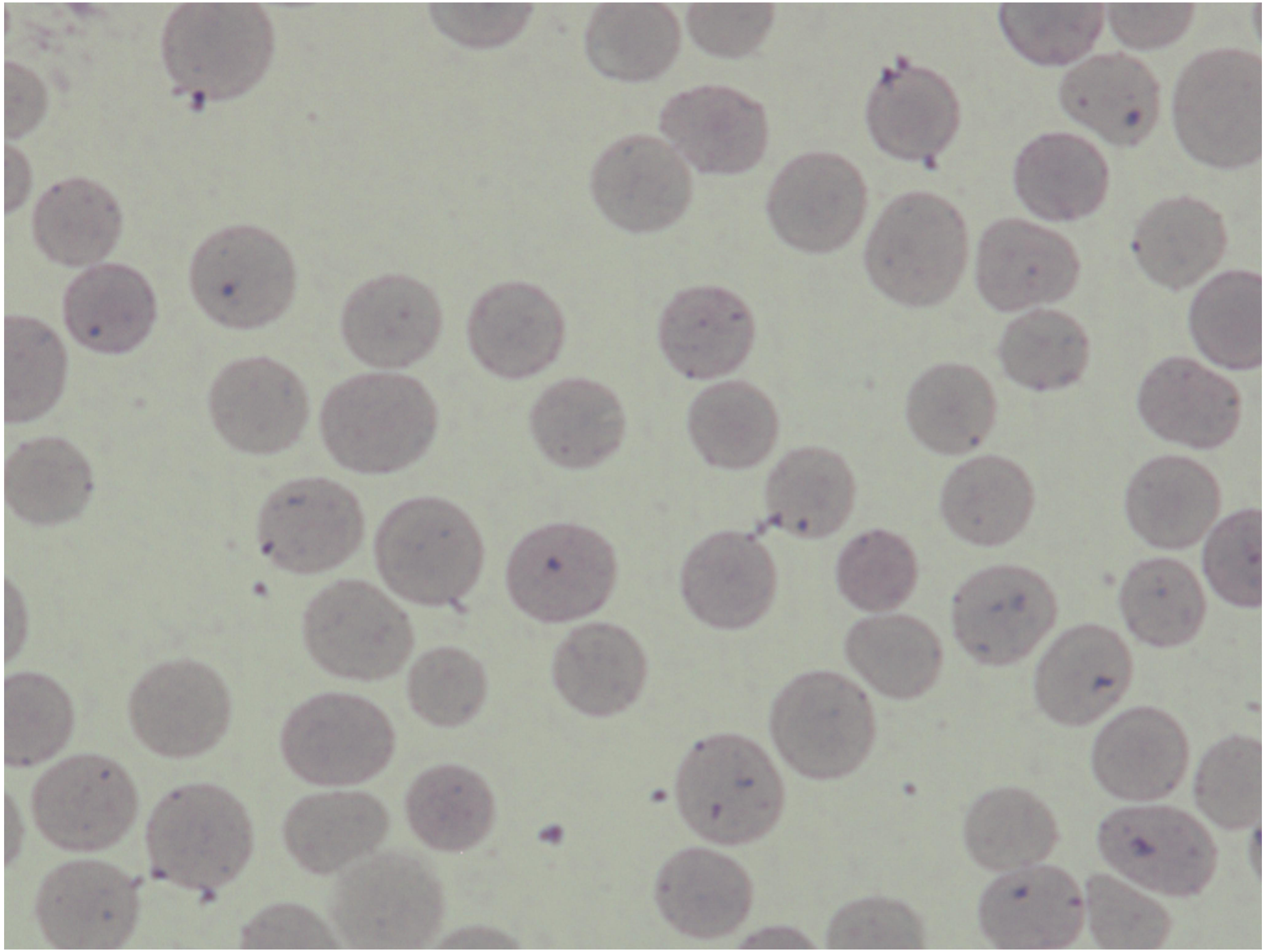

Figure S1 1x RO
72h （200×10）

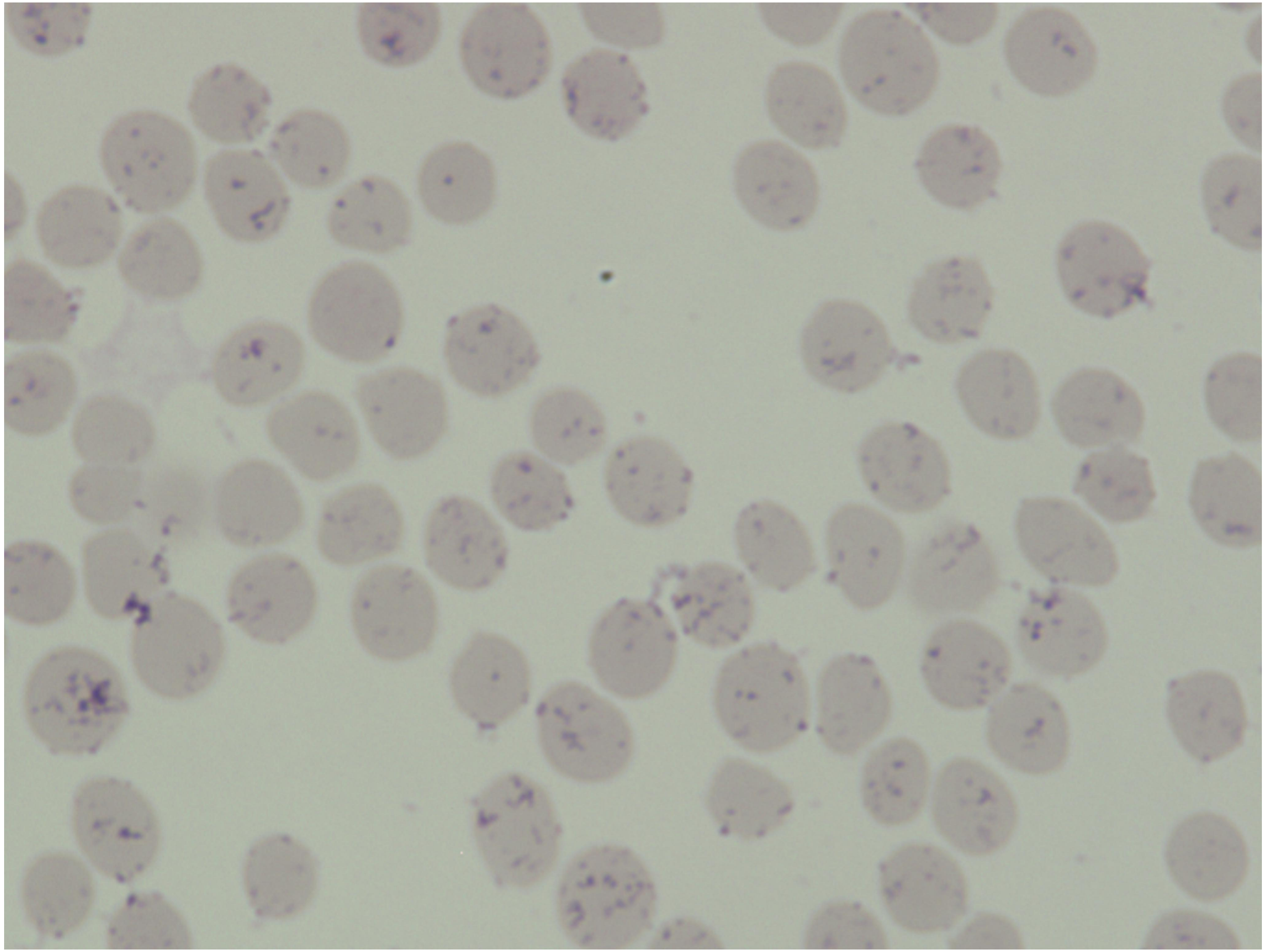

Figure S1 1x RO 7d
（200×10）

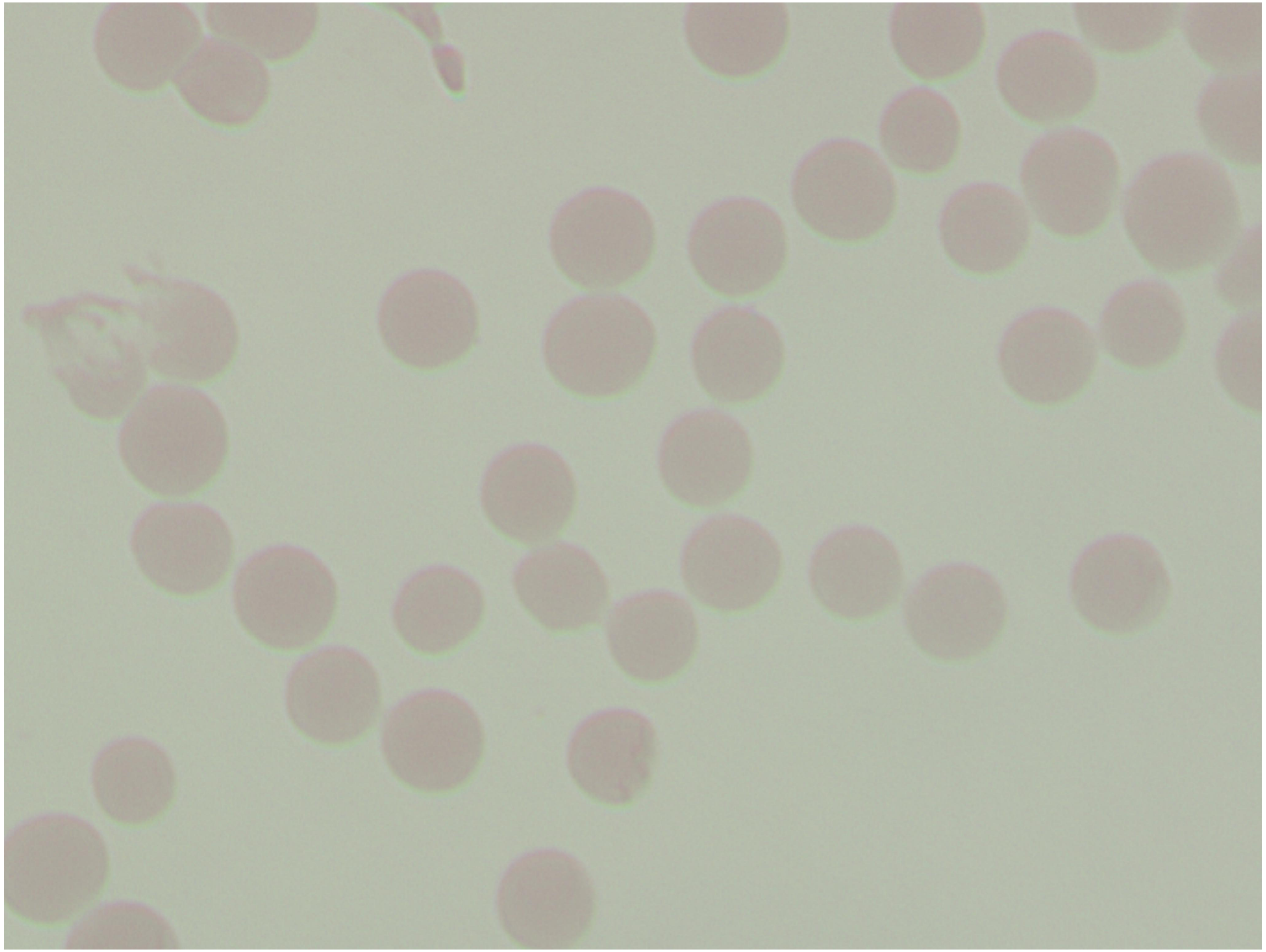

Figure S1 2x RO
24h （200×10）

Figure S1 2x RO
72h （200×10）

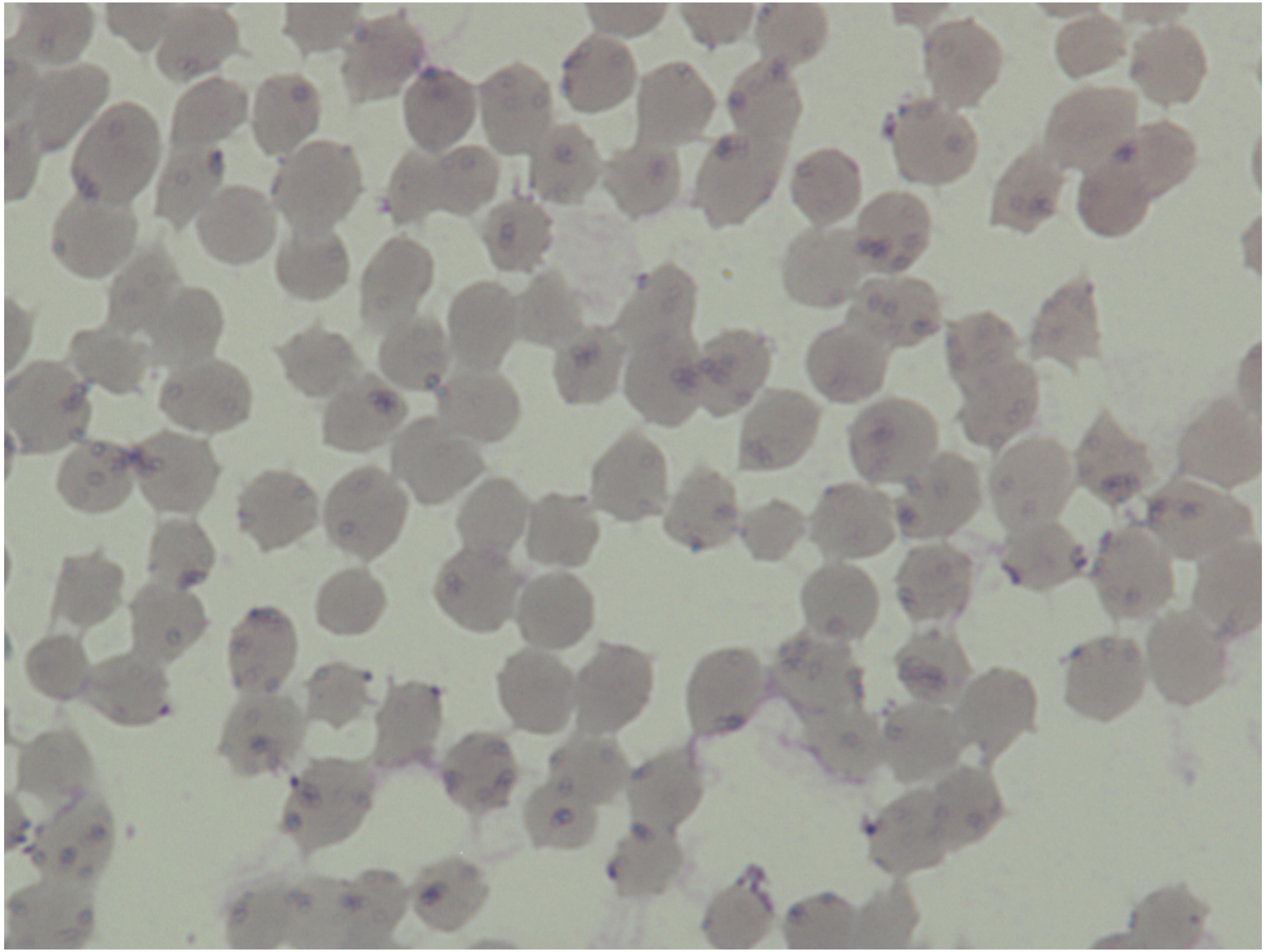

Figure S1 2x
RO 7d

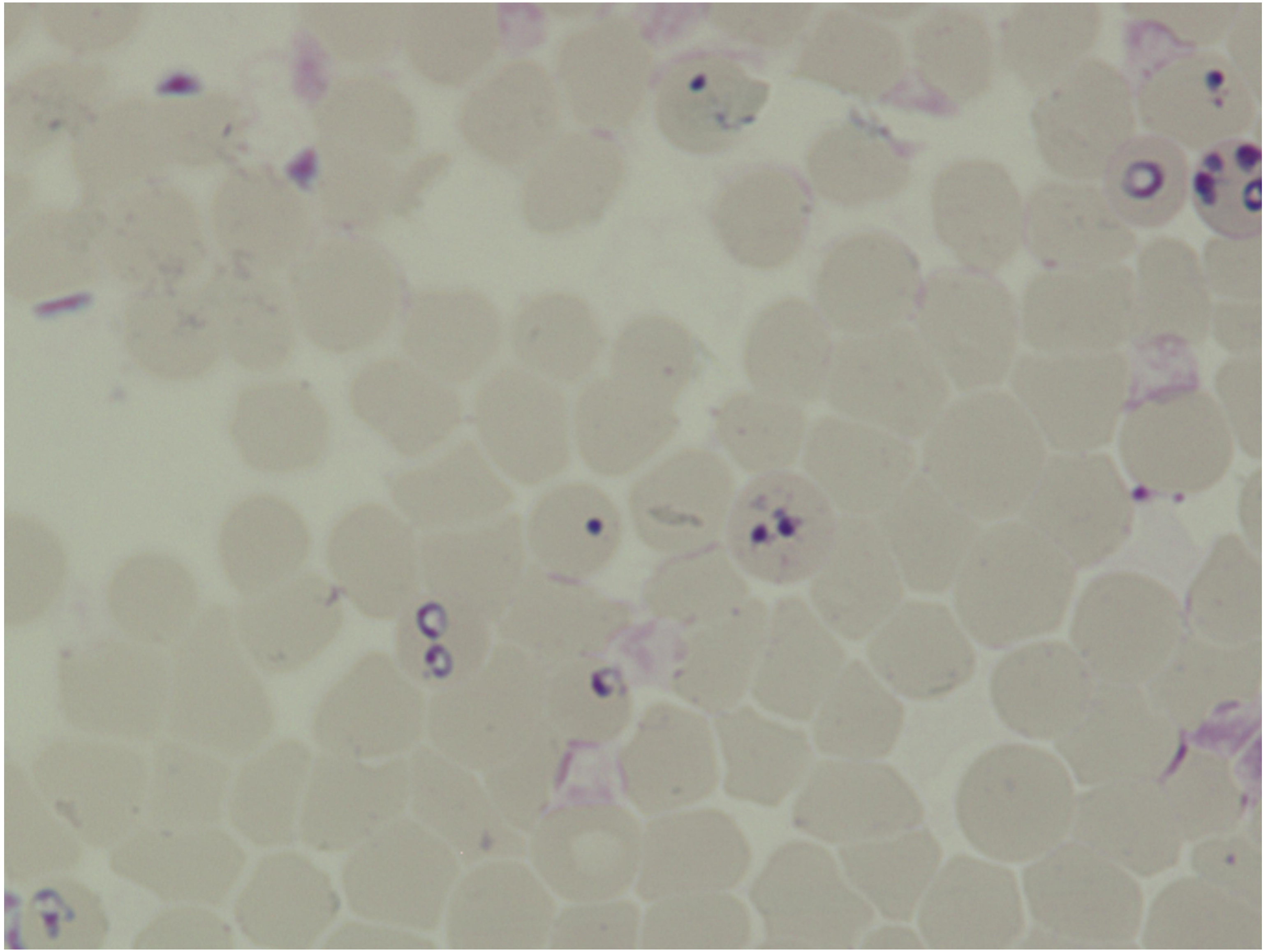

Figure S2
Positive P3d
（200×10）

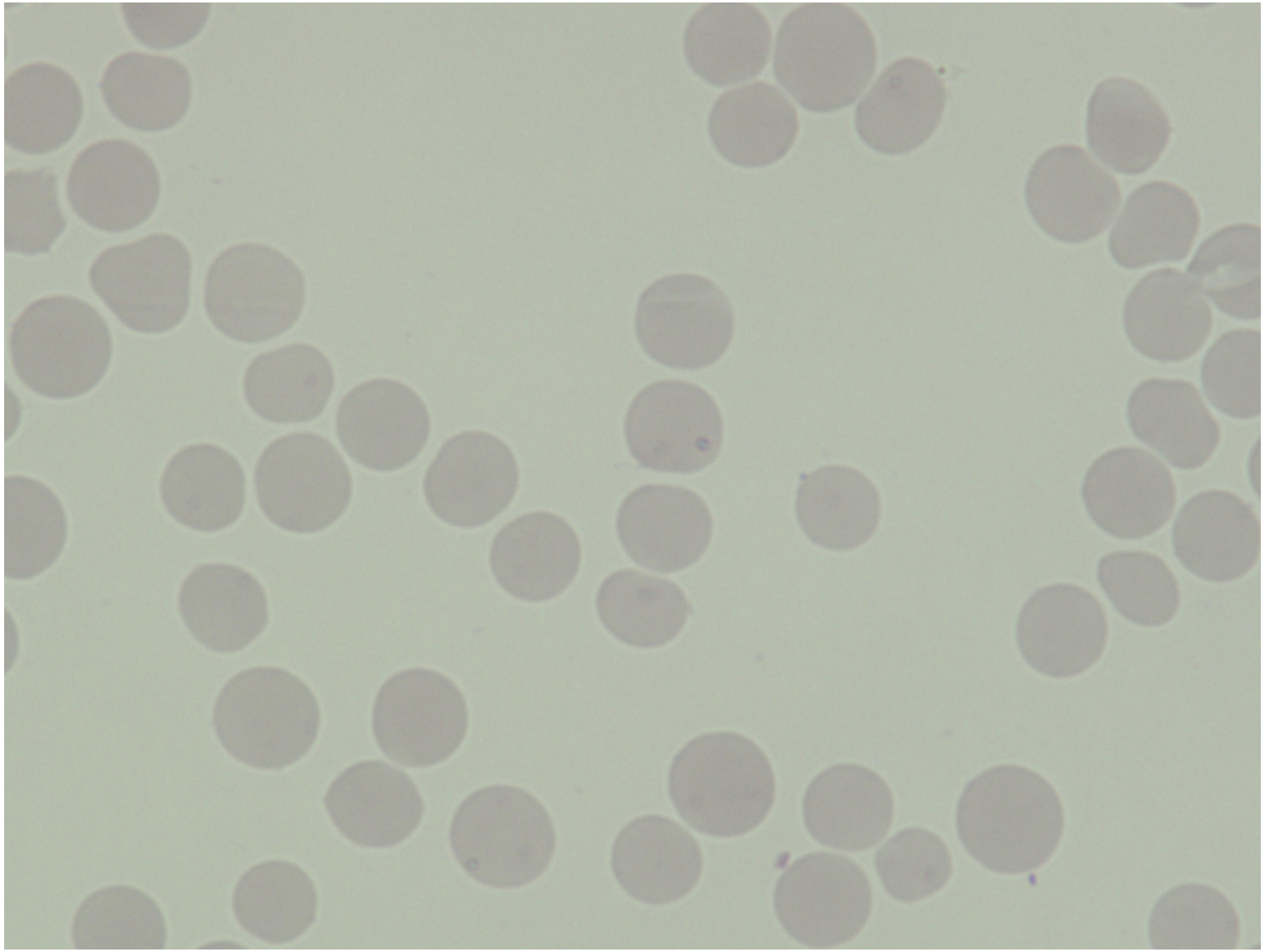

Figure S2 0.5x
NR 24h

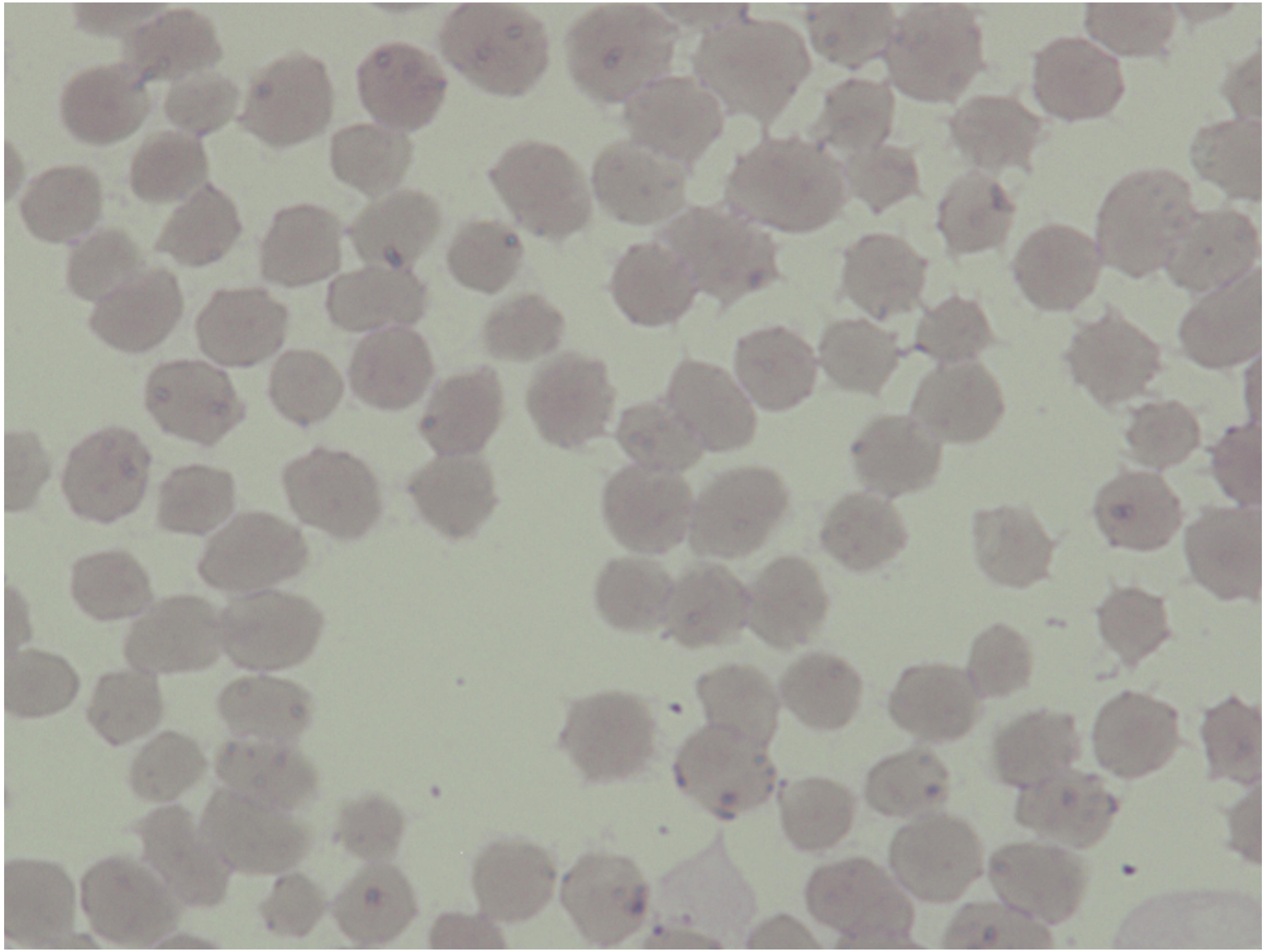

Figure S2 0.5x
NR 72h

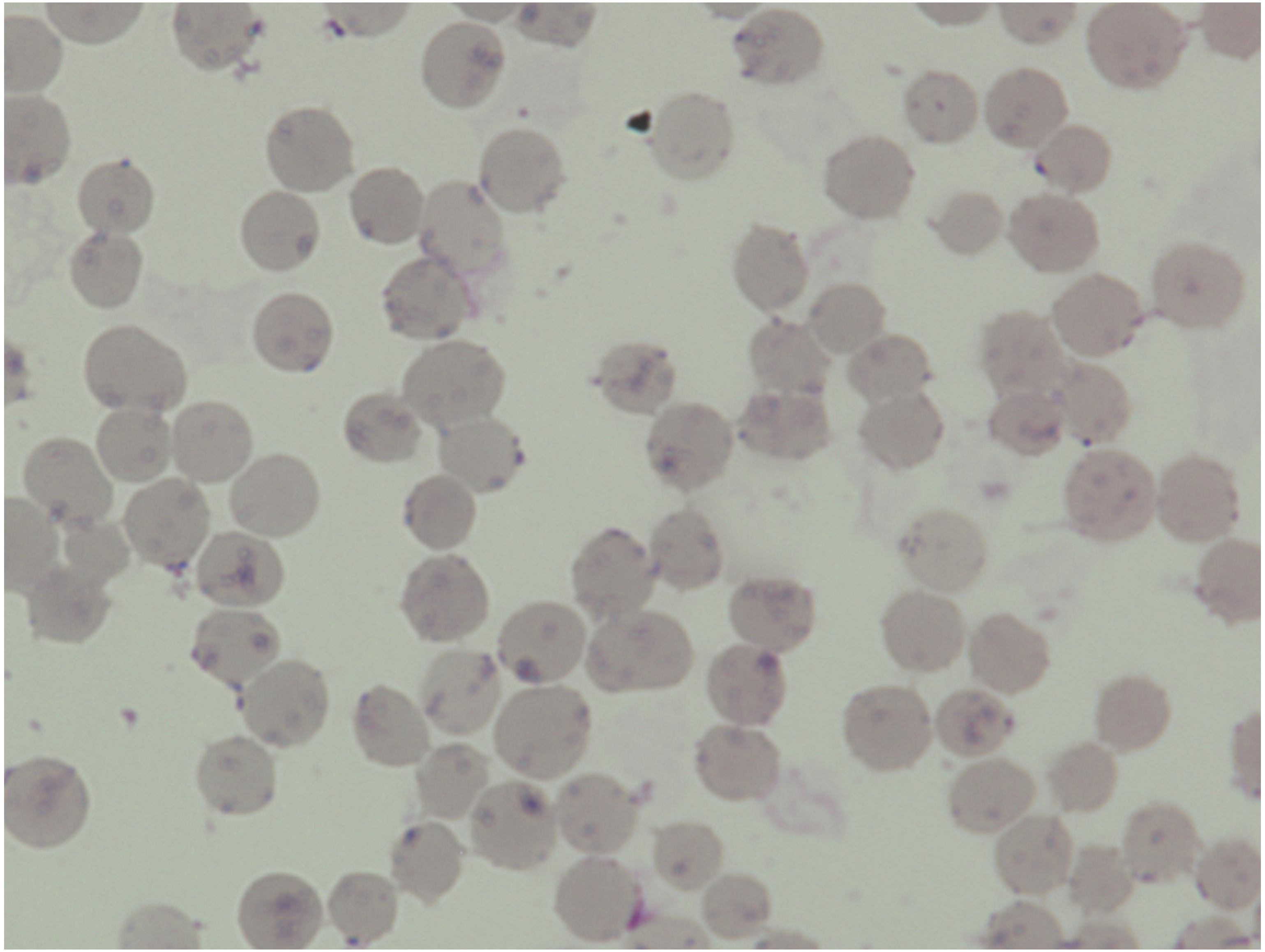

Figure S2 0.5x
NR 7d

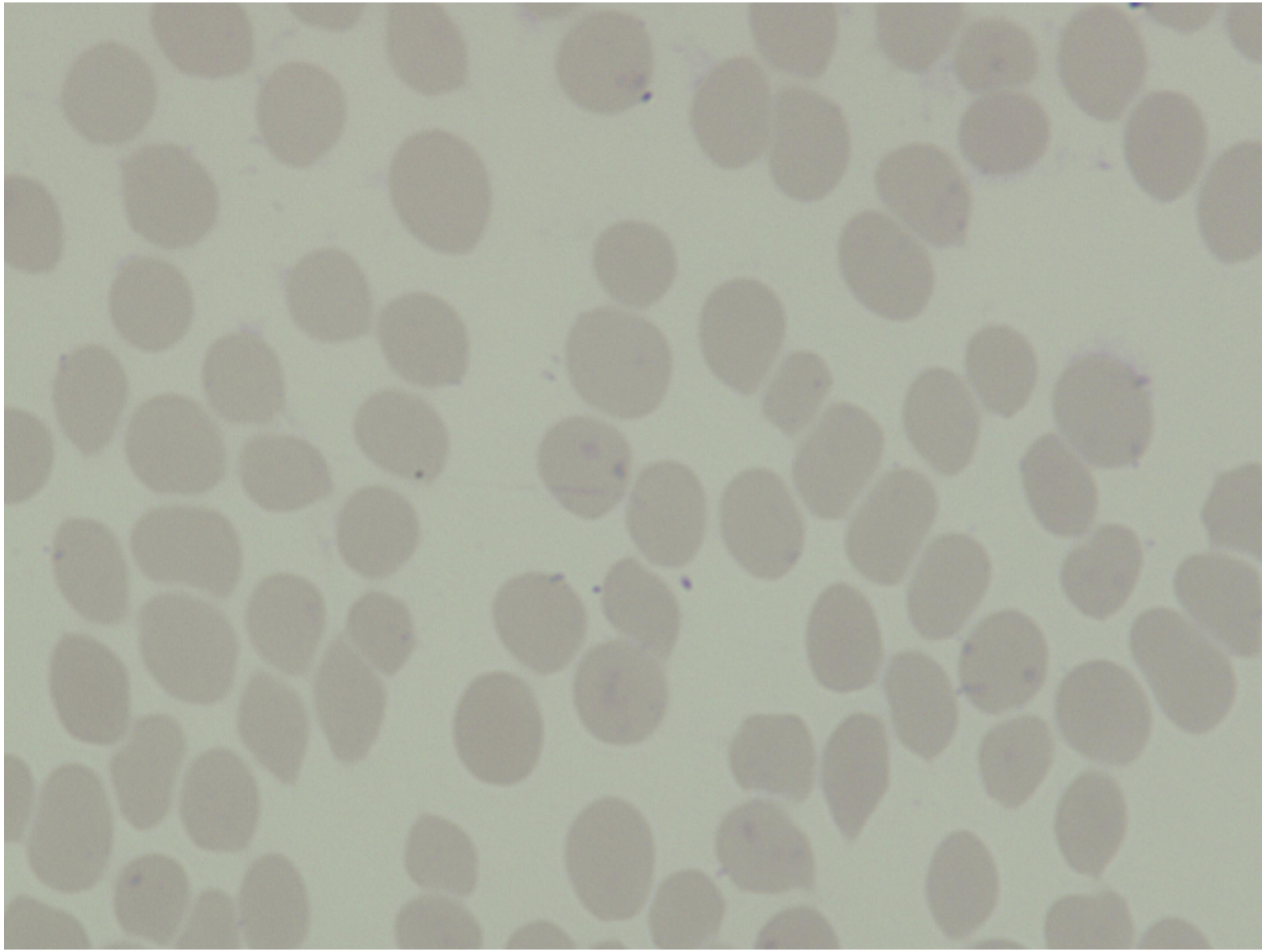

Figure S2 1x
NR 24h

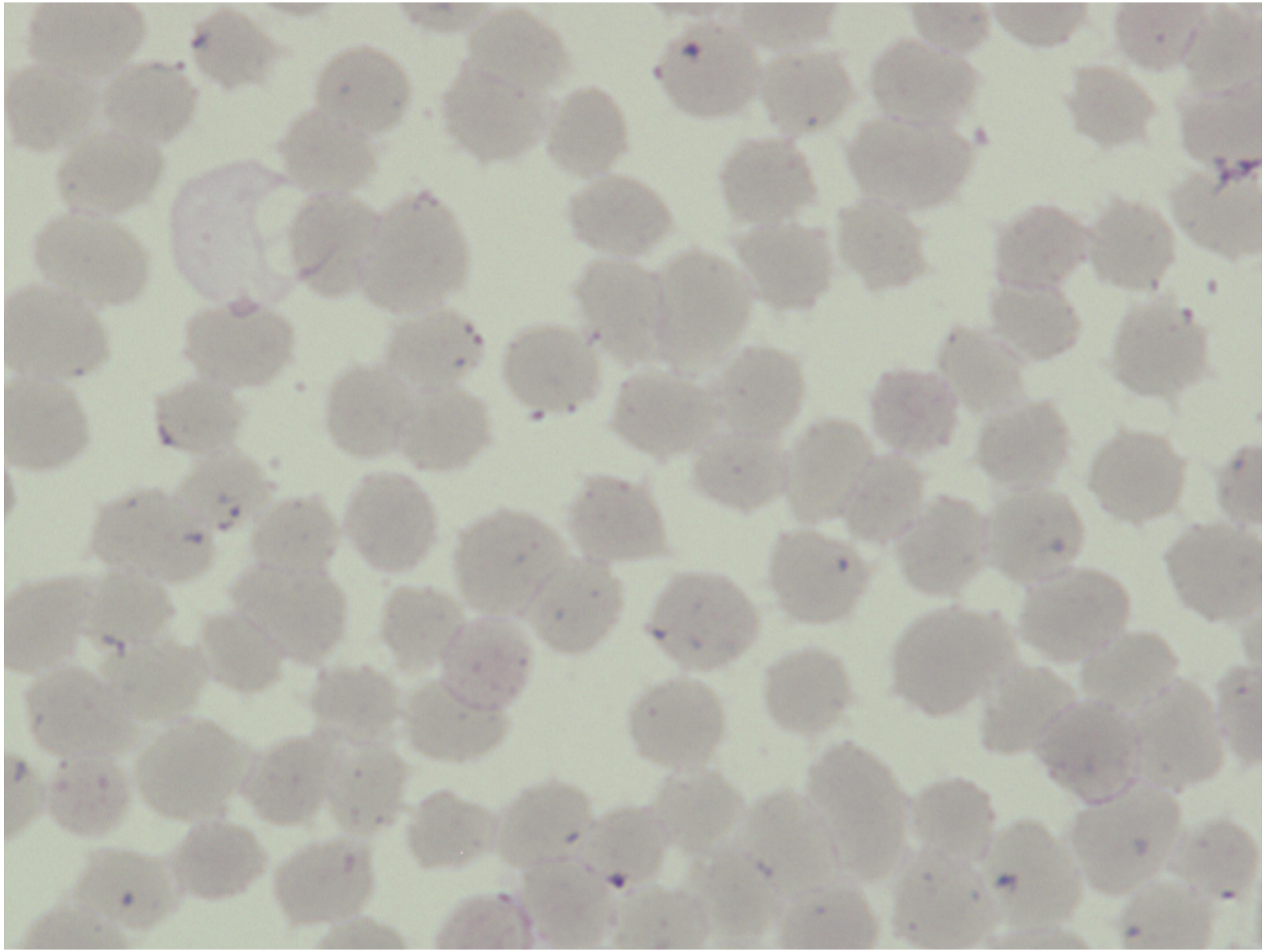

Figure S2 1x
NR 72h

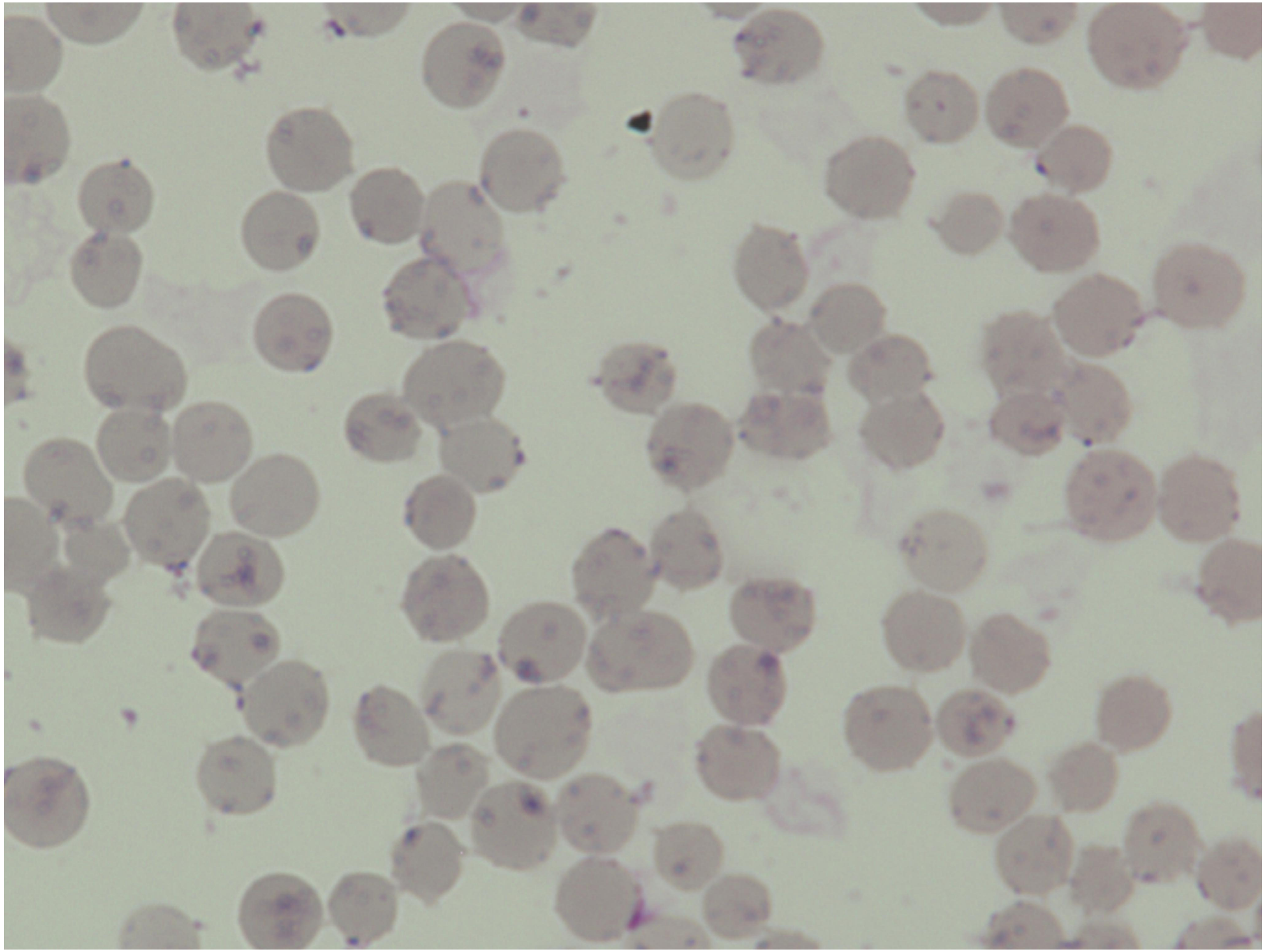

Figure S2 1x
NR 7d

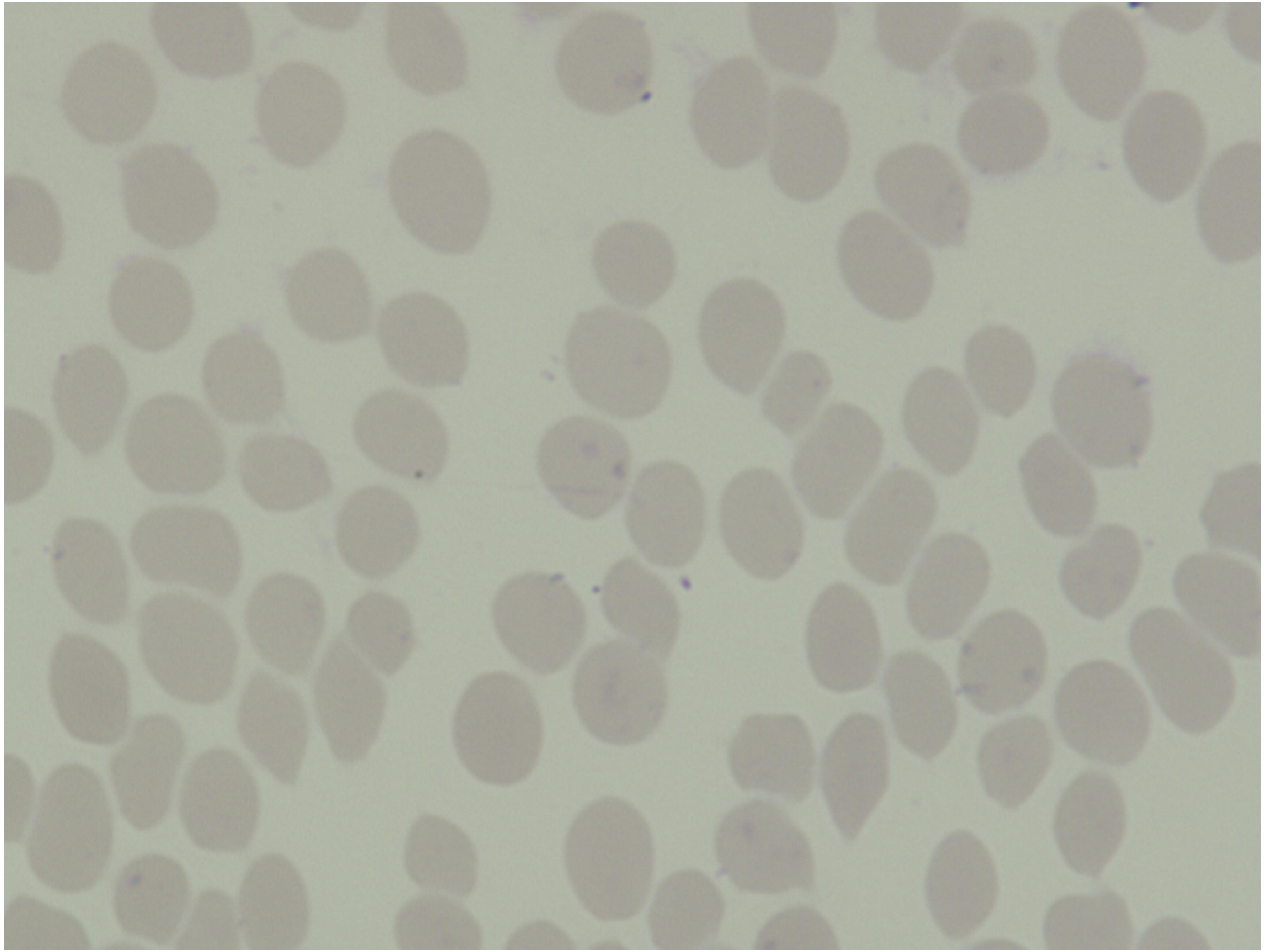

Figure S2 2x
NR 24h

Figure S2 2x
NR 72h

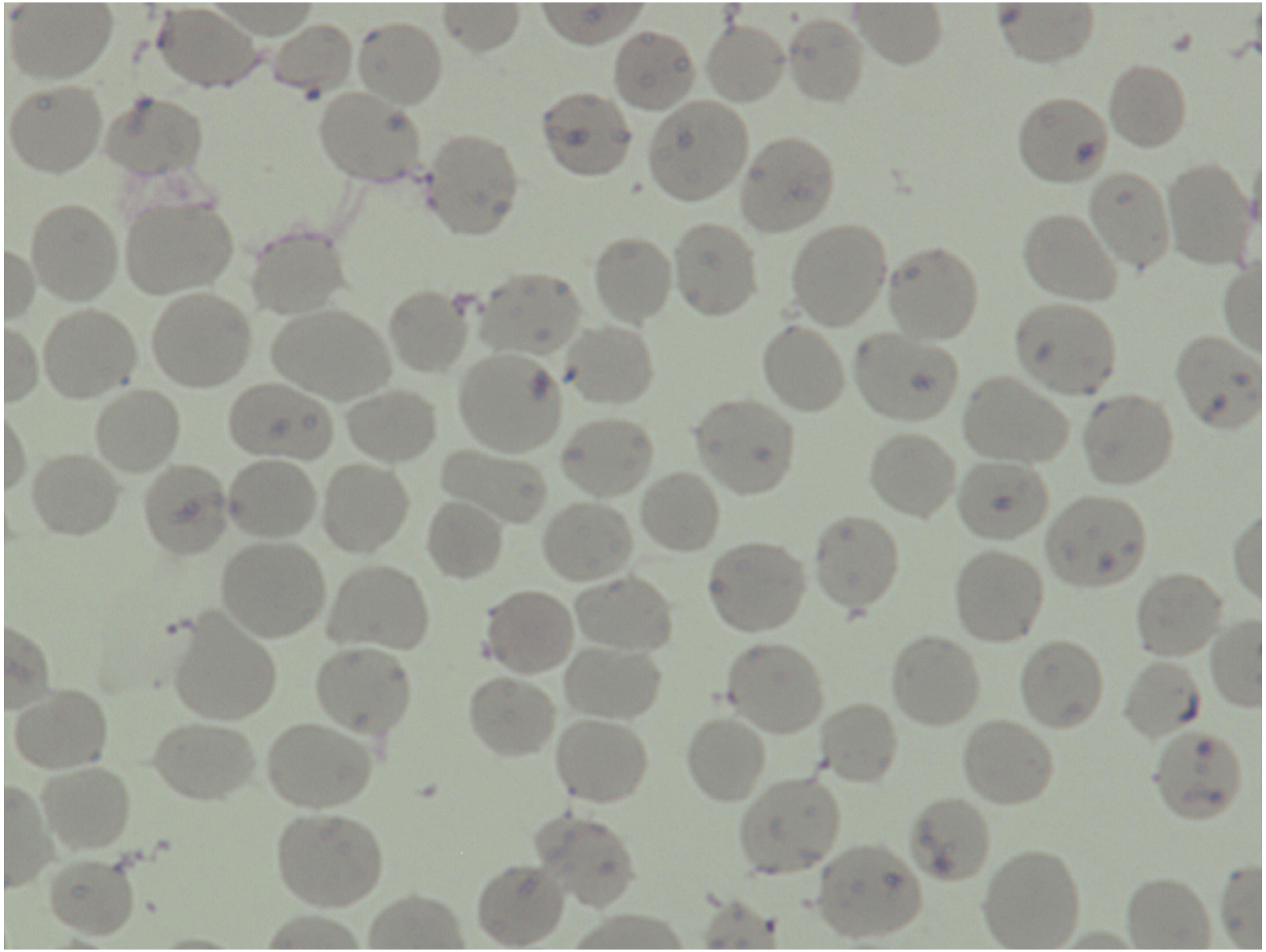

Figure S2 2x
NR 7d

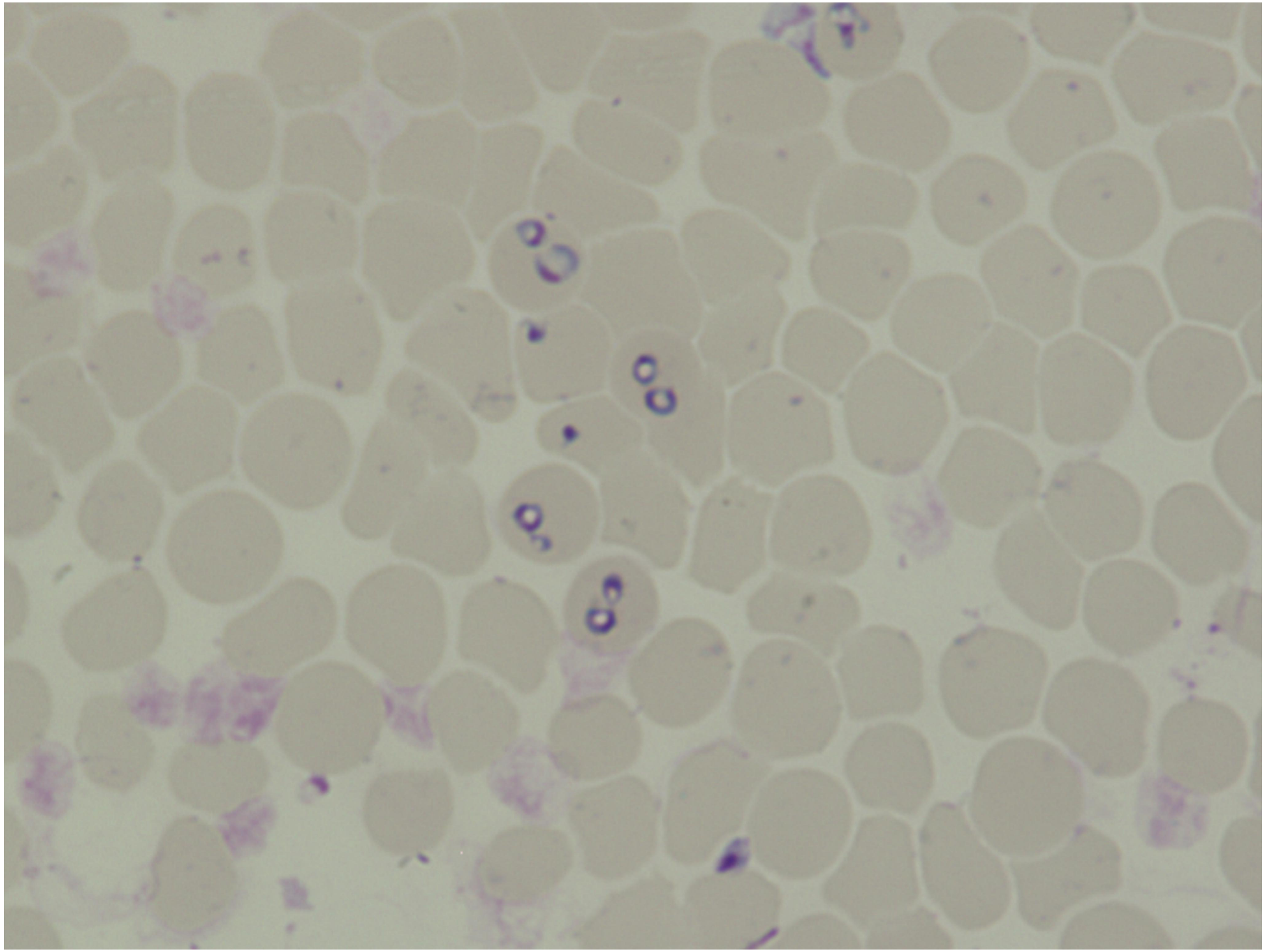

Figure S3
Positive P3d
（200×10）

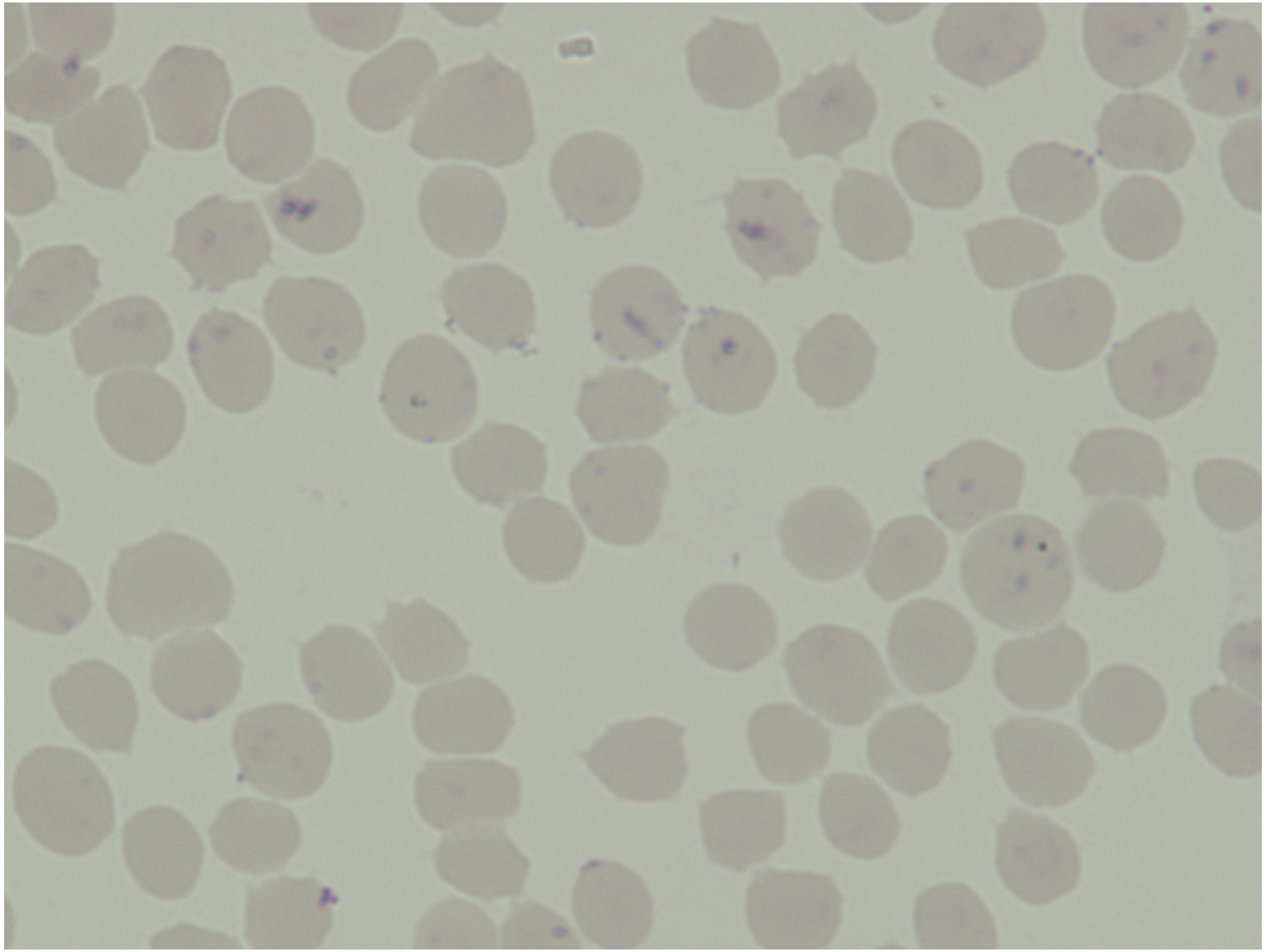

Figure S3 0.5x
LA 24h

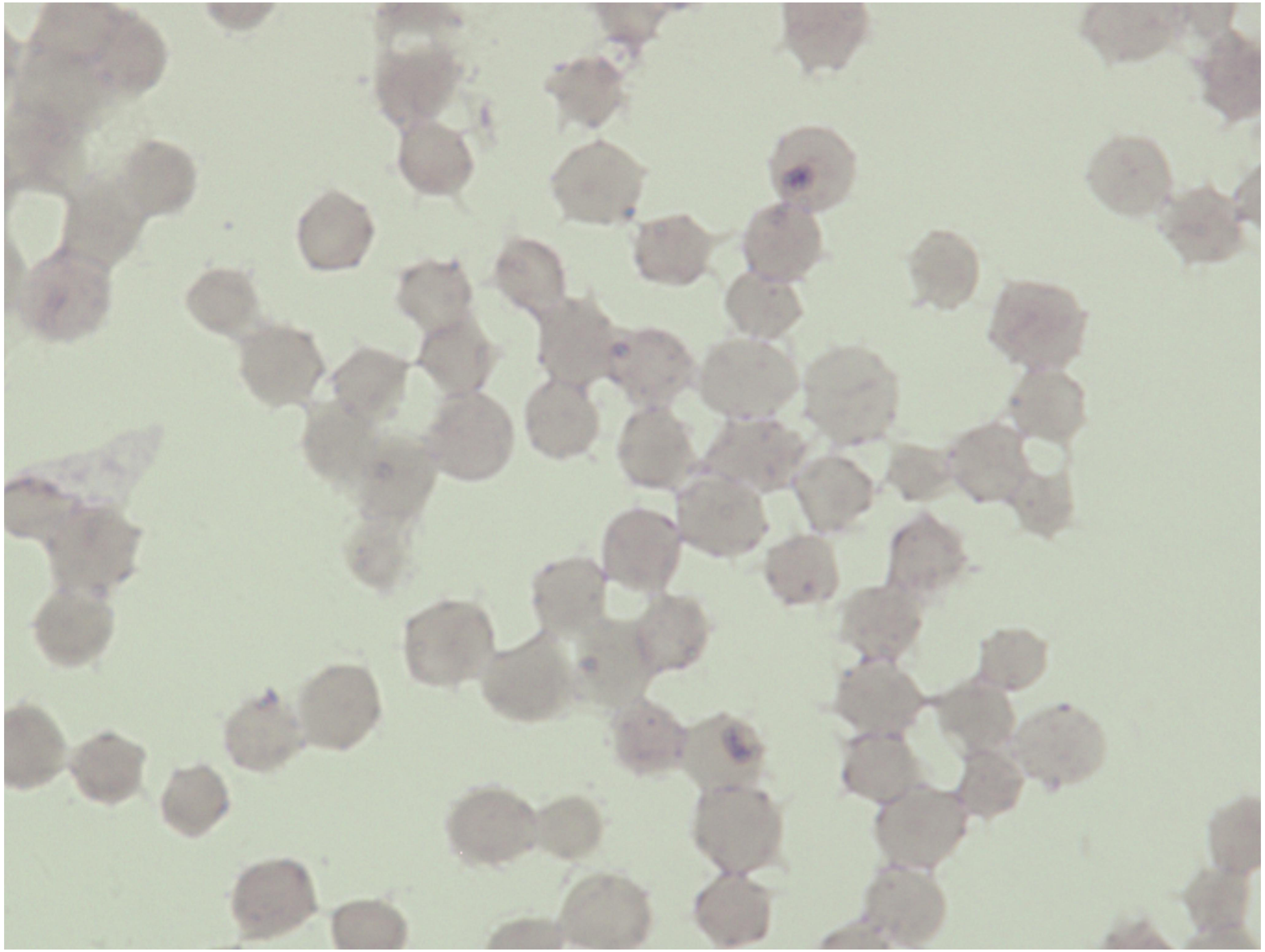

Figure S3 0.5x
LA 72h

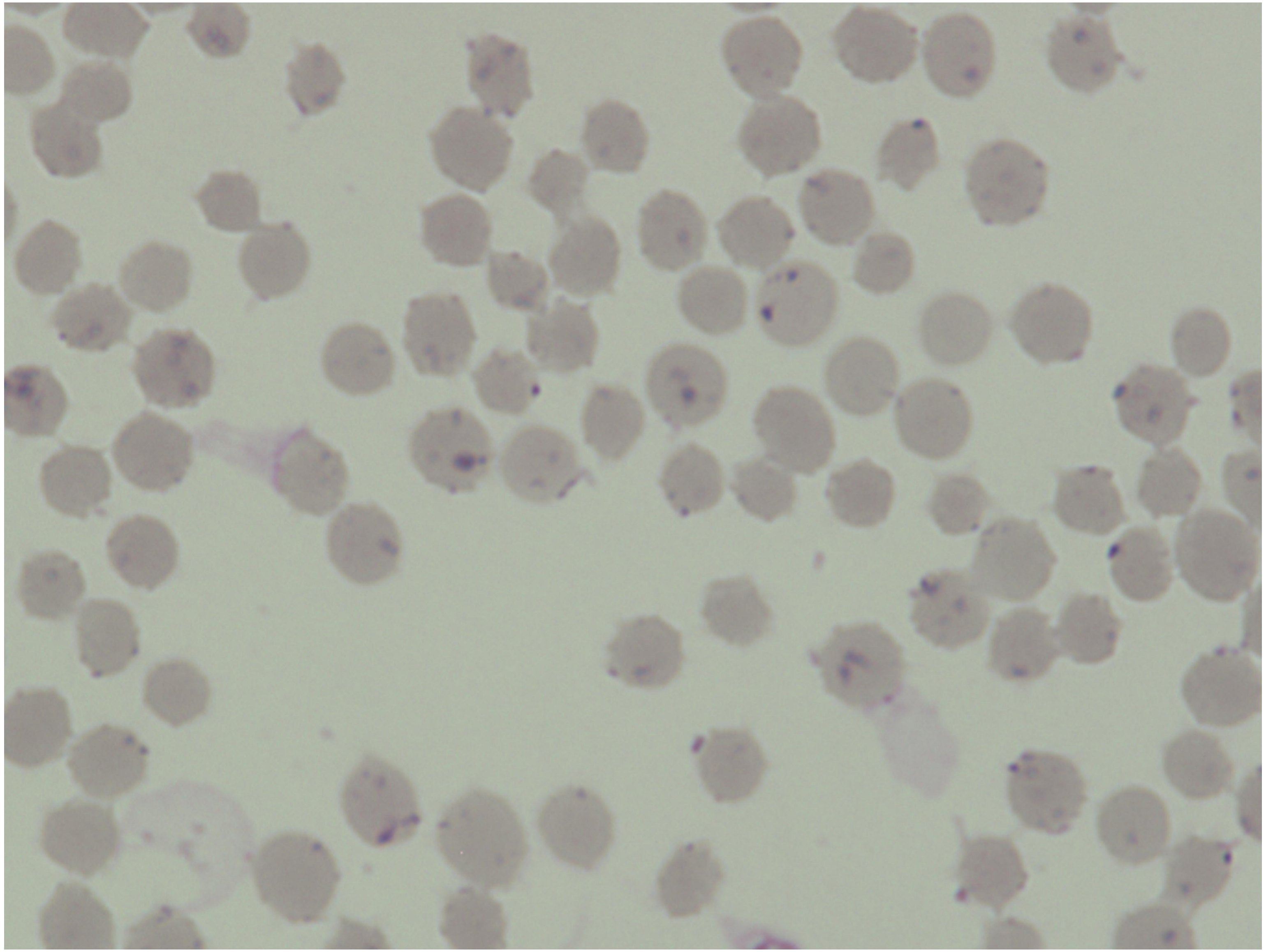

Figure S3 0.5x
LA 7d

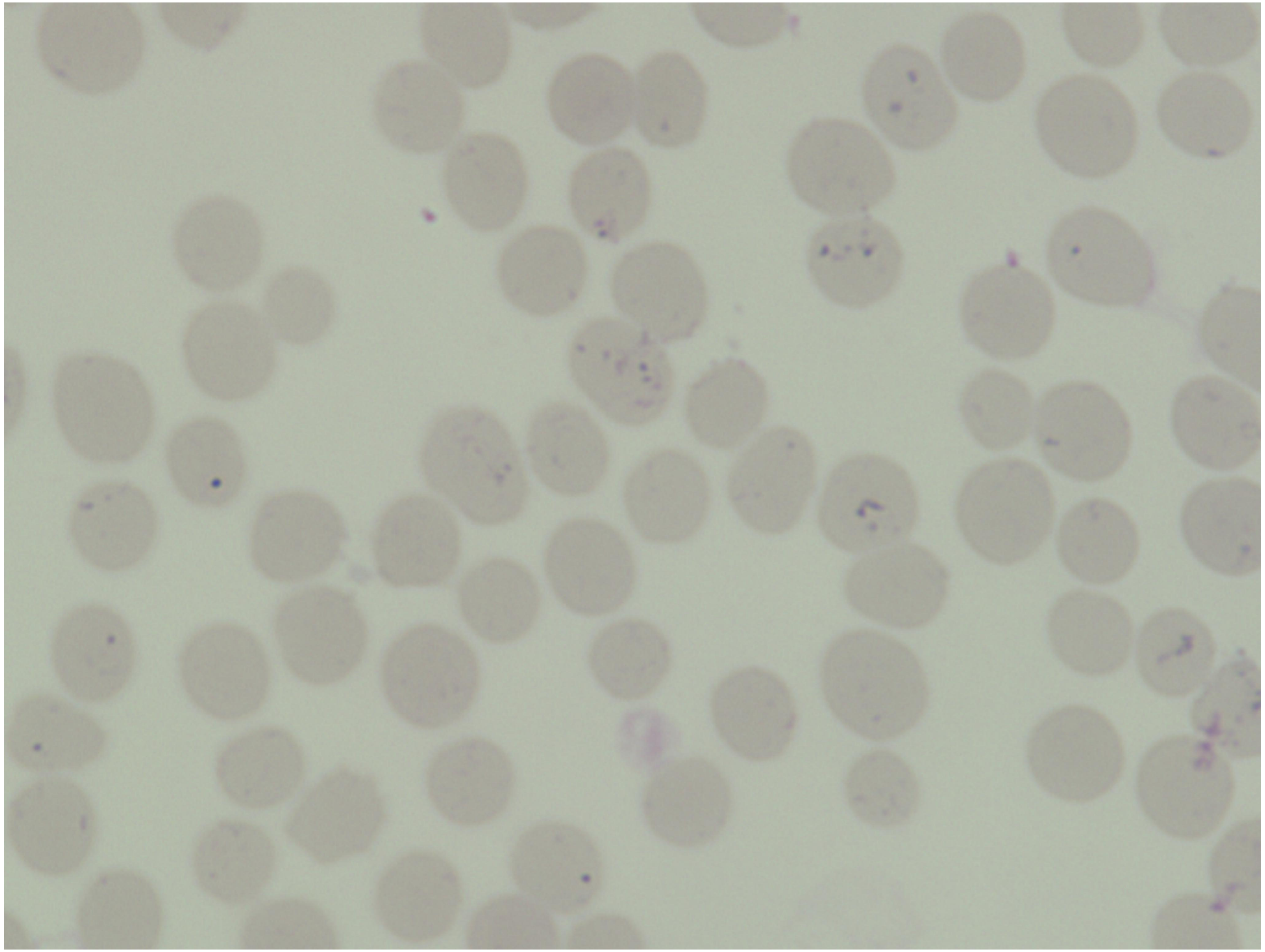

Figure S3 1x
LA 24h

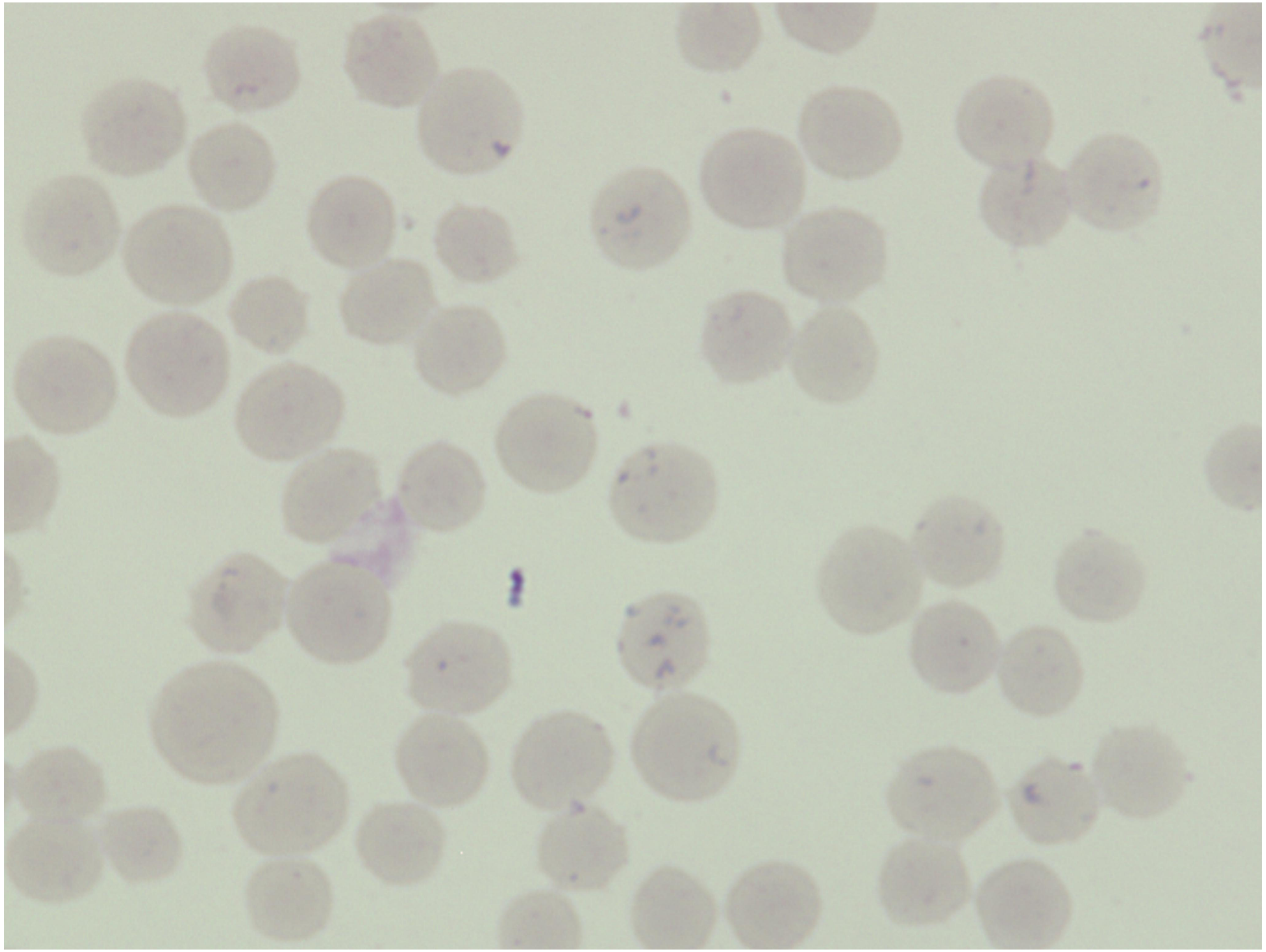

Figure S3 1x
LA 72h

Figure S3 1x
LA 7d

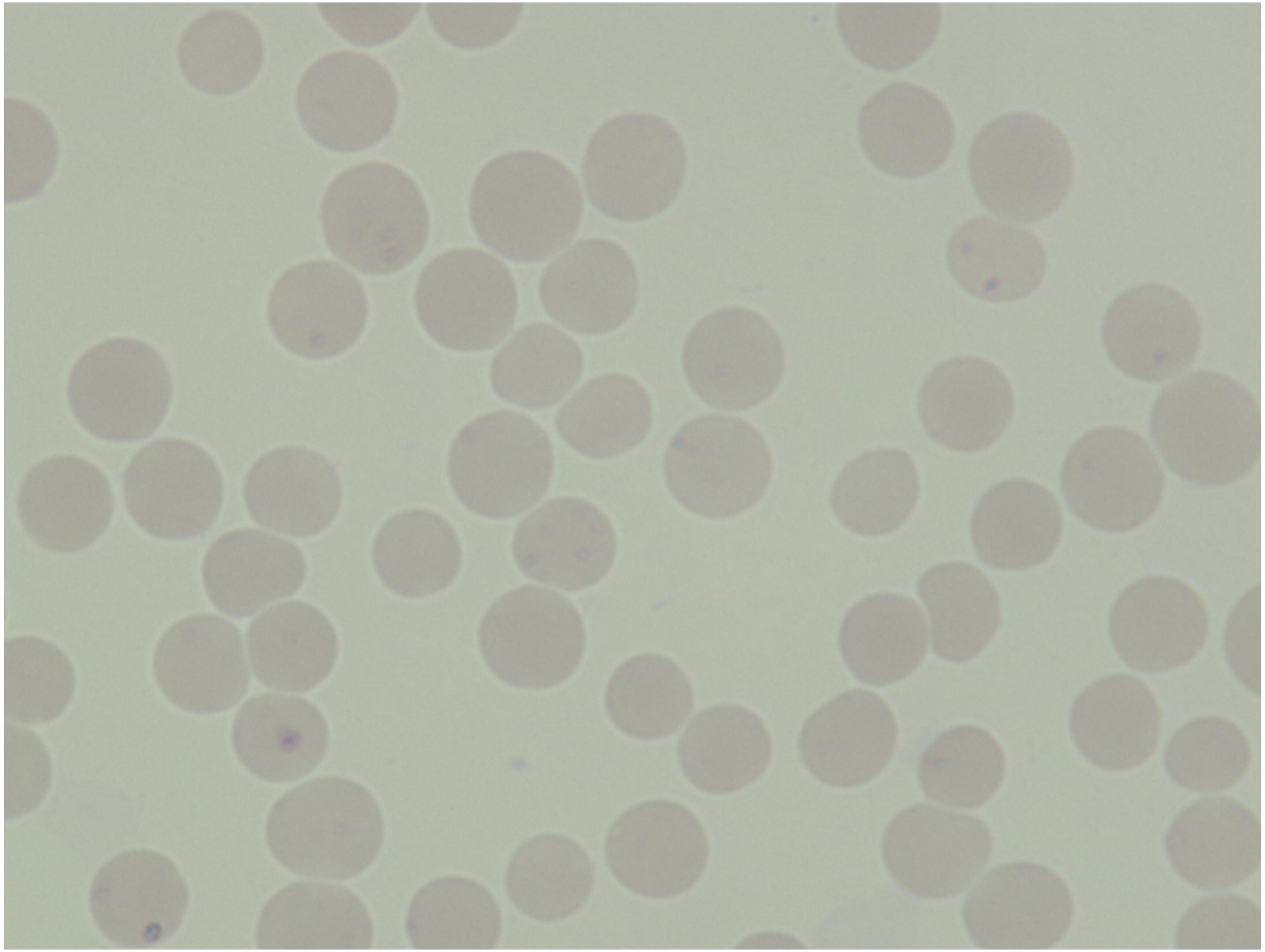

Figure S3 2x
LA 24h

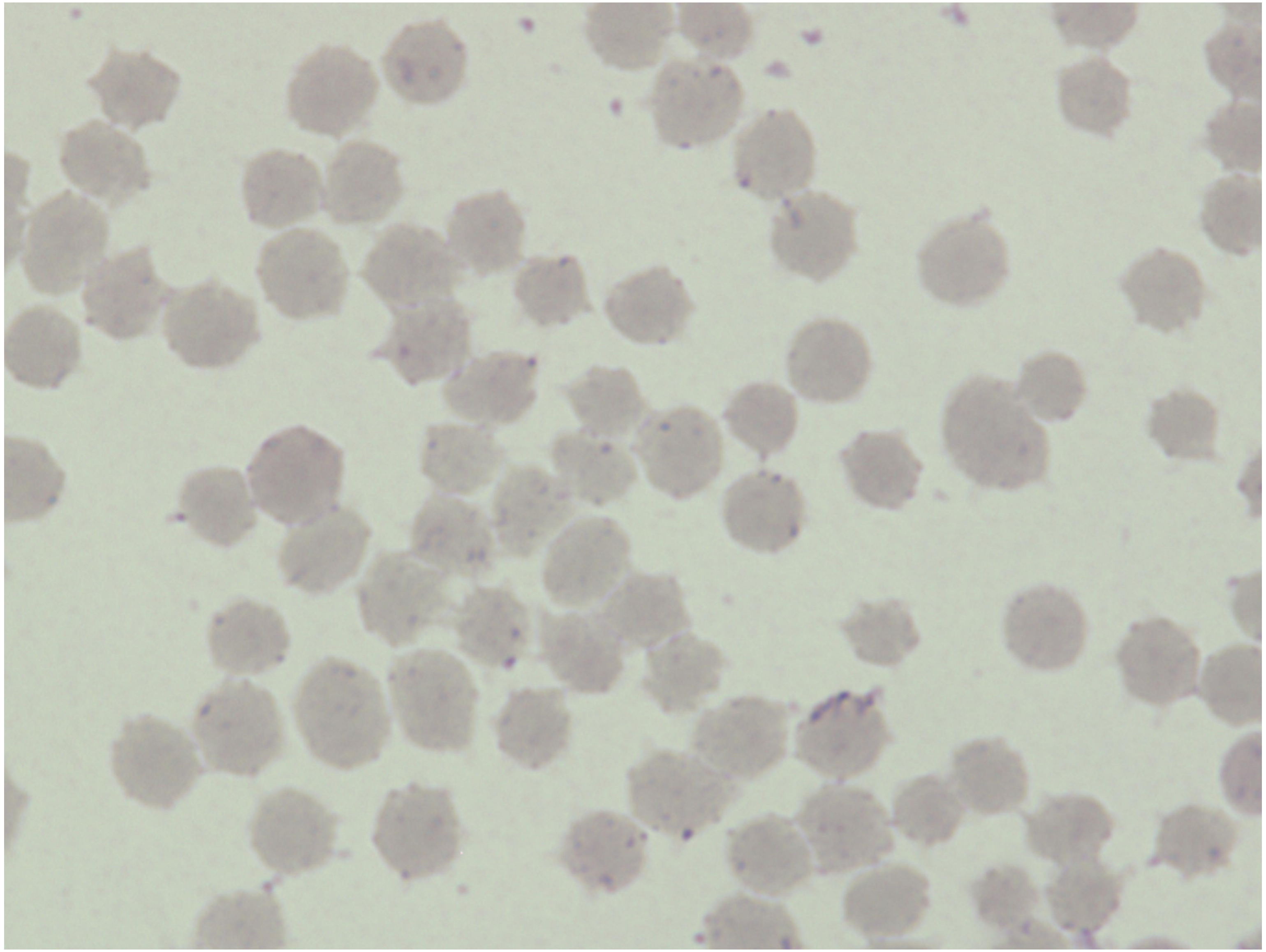

Figure S3 2x
LA 72h

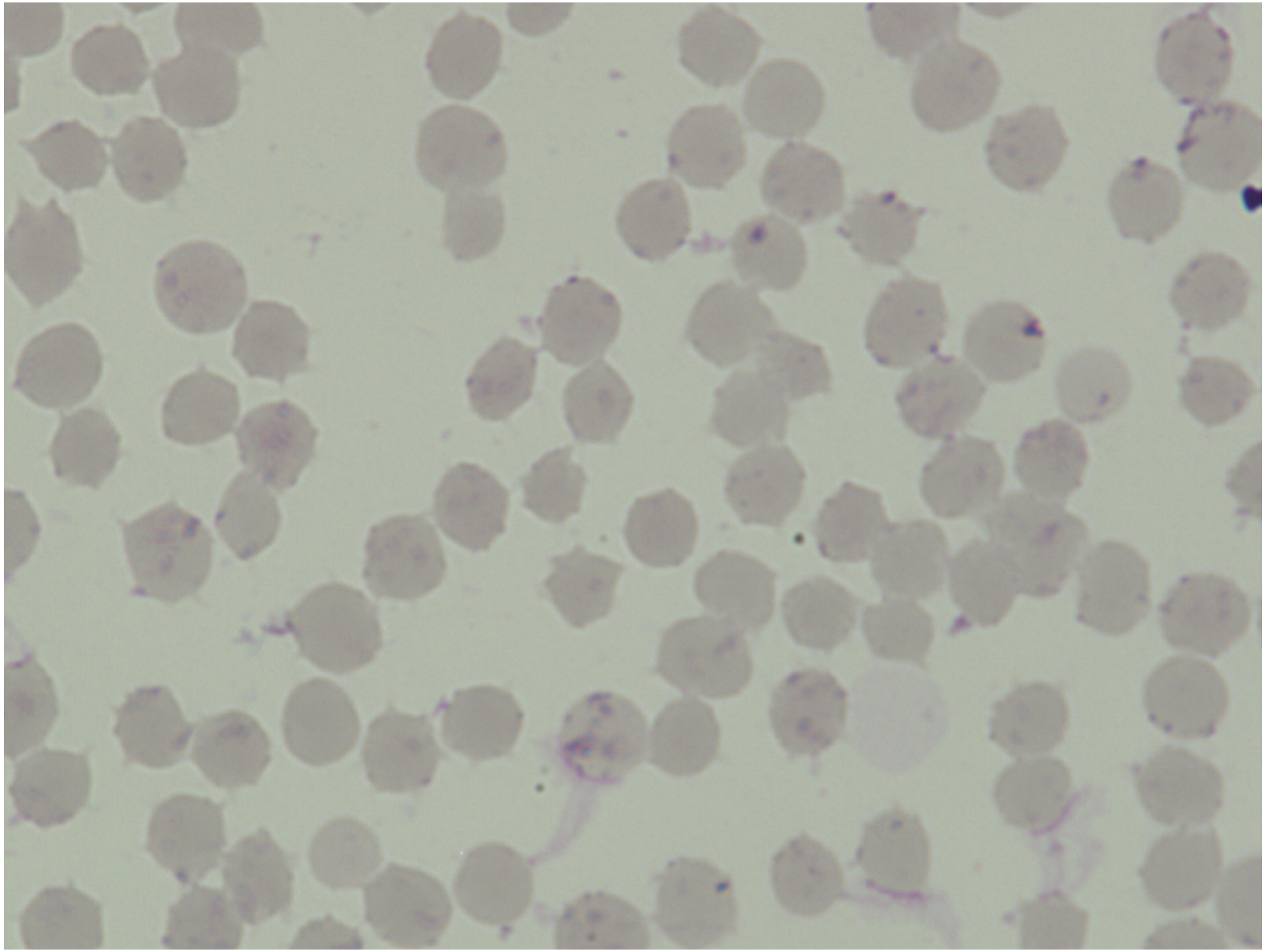

Figure S3 2x
LA 7d
