# Supplementary figures and images for: Discovering the Potent Inhibitors Against Babesia bovis in vitro and Babesia microti in vivo by Repurposing the Natural Product Compounds
Source: Front Vet Sci. 2021 Nov 29;8:762107. doi: 10.3389/fvets.2021.762107 (PMC8666878; doi:10.3389/fvets.2021.762107)

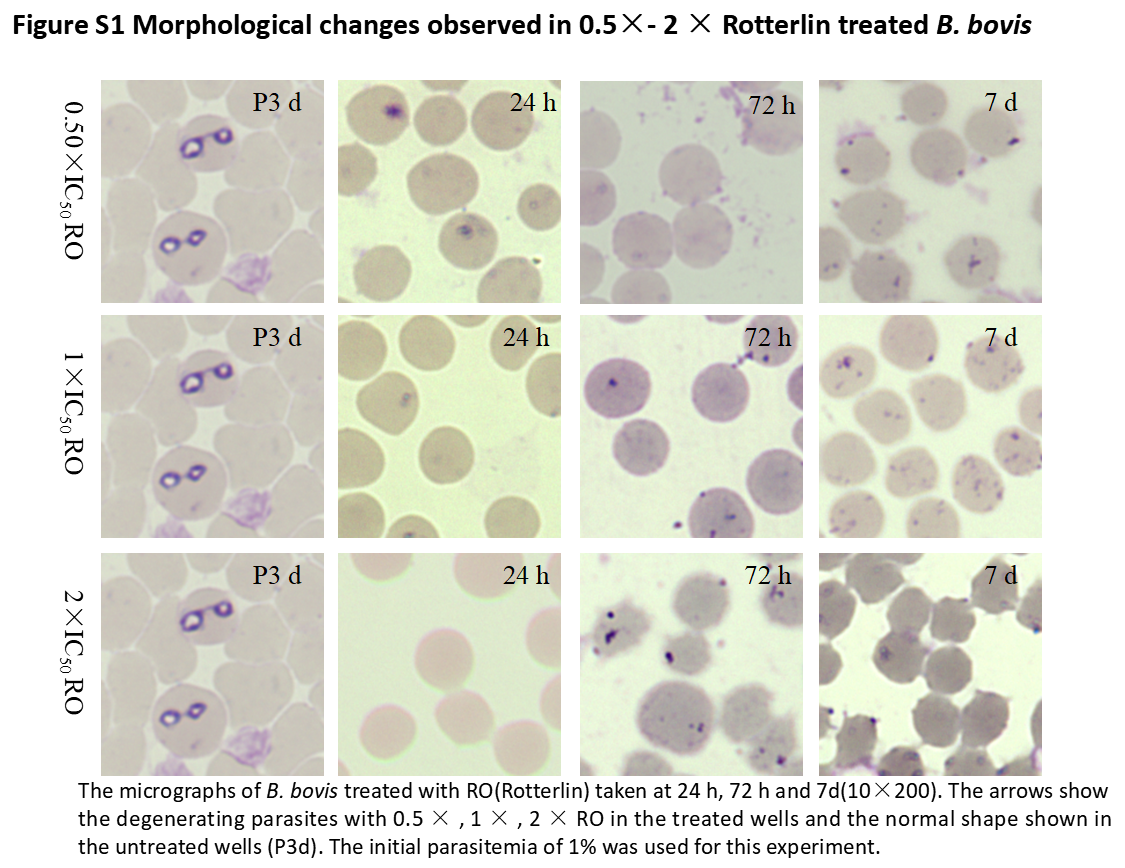

Supplement: Supplementary Figure 1 — Morphological changes observed in 0.5 × -2 × Rotterolin treated B. bovis. [file Image_1.tif]

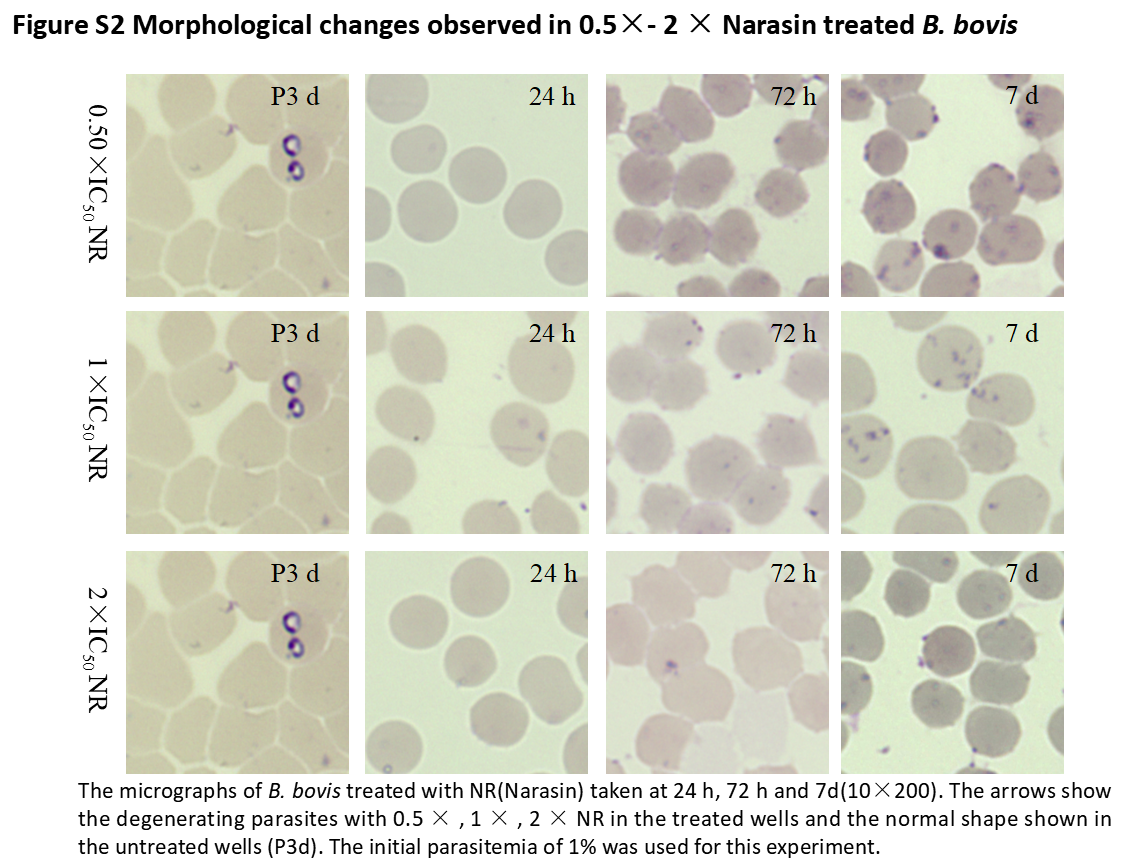

Supplement: Supplementary Figure 2 — Morphological changes observed in 0.5 × -2 × Narasin treated B. bovis. [file Image_2.tif]

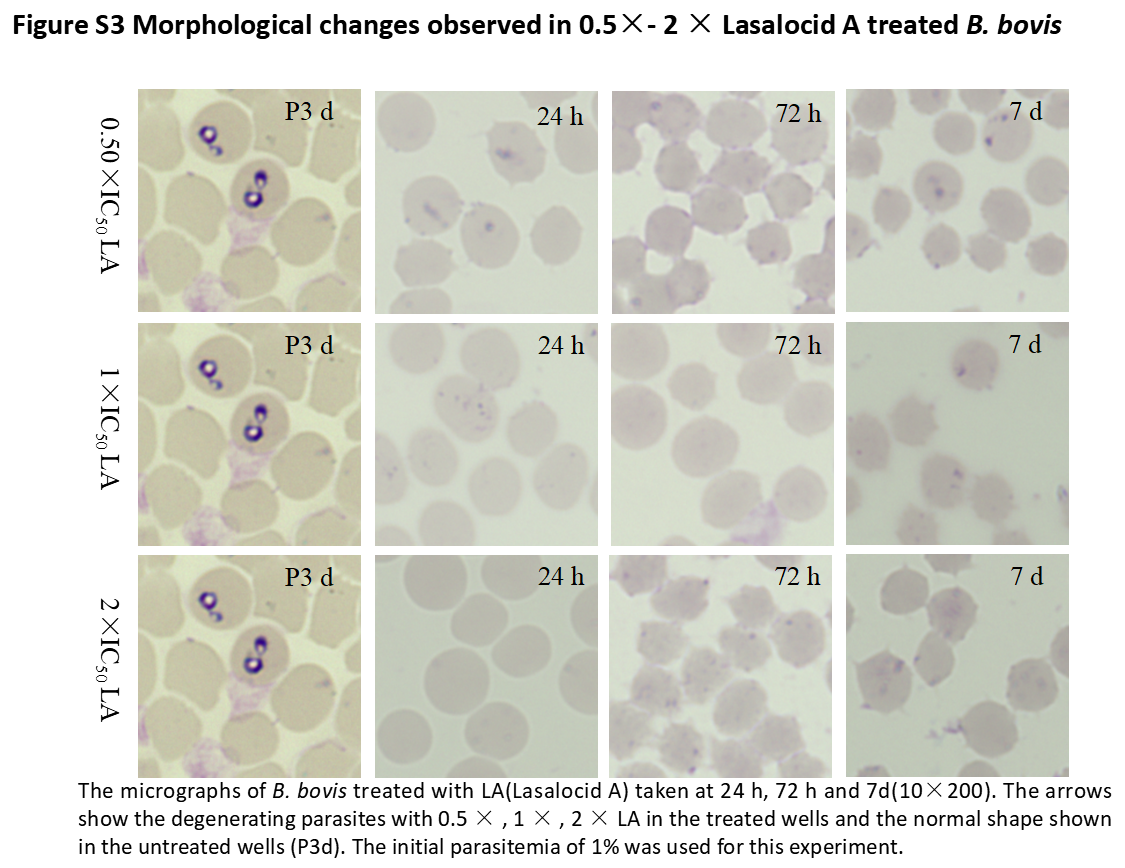

Supplement: Supplementary Figure 3 — Morphological changes observed in 0.5 × -2 × Lasalocid A treated B. bovis. [file Image_3.tif]

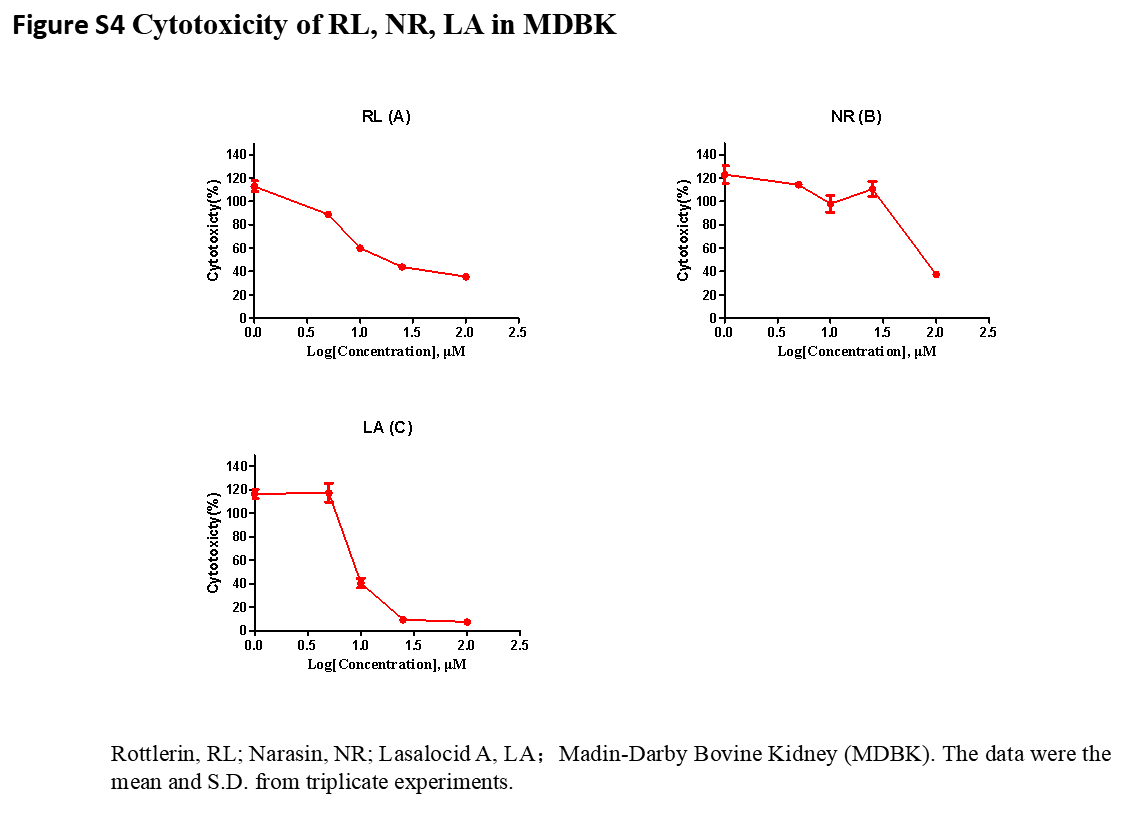

Supplement: Supplementary Figure 4 — Cytotoxicity of RL, NR, and LA in MDBK. [file Image_4.tif]
